# 3D printed scaffolds with multistage osteogenic activity for bone defect repair

**DOI:** 10.1093/rb/rbaf010

**Published:** 2025-03-10

**Authors:** Bing Li, Yichao Ma, Kanwal Fatima, Xiaojun Zhou, Shuo Chen, Chuanglong He

**Affiliations:** State Key Laboratory for Modification of Chemical Fibers and Polymer Materials, College of Biological Science and Medical Engineering, Donghua University, Shanghai 201620, China; Department of Orthopedics, Shanghai General Hospital, Shanghai Jiao Tong University School of Medicine, Shanghai 200080, China; State Key Laboratory for Modification of Chemical Fibers and Polymer Materials, College of Biological Science and Medical Engineering, Donghua University, Shanghai 201620, China; State Key Laboratory for Modification of Chemical Fibers and Polymer Materials, College of Biological Science and Medical Engineering, Donghua University, Shanghai 201620, China; State Key Laboratory for Modification of Chemical Fibers and Polymer Materials, College of Biological Science and Medical Engineering, Donghua University, Shanghai 201620, China; State Key Laboratory of Molecular Engineering of Polymers, Fudan University, Shanghai 200433, China; State Key Laboratory for Modification of Chemical Fibers and Polymer Materials, College of Biological Science and Medical Engineering, Donghua University, Shanghai 201620, China

**Keywords:** multistage osteogenic activity, 3D printing, immune microenvironment, bone repair

## Abstract

The bone defect repair is a complex process including immune regulation, stem cell osteogenic differentiation and extracellular matrix mineralization. Current bone tissue engineering approaches often fail to adapt throughout the above osteogenic process, resulting in suboptimal repair outcomes. To address this problem, a 3D-printed scaffold with multistage osteogenic activity based on shape-memory elastomer and electroactive material is developed. The scaffold exhibits excellent shape memory performance and can trigger shape recovery by physiological temperature. The physiological temperature-triggered shape-memory behavior makes the scaffold promising for minimally invasive implantation. After electric field polarization, the scaffold’s surface carries the negative charge, which can activate the PI3K/Akt signaling pathway to promote the polarization of macrophages to M2 phenotype and activate the FAK/ERK signaling pathway to promote osteogenic differentiation of bone marrow mesenchymal stem cells (BMSCs), indicating that the scaffold can effectively participate in immune microenvironment regulation and stem cell osteogenic differentiation. Additionally, the negative charge on the scaffold’s surface can attract calcium and phosphate ions, forming a mineralized matrix and promoting late-stage extracellular matrix mineralization by continuously supplying mineralizing ions such as calcium and phosphate. Overall, this study introduces a 3D-printed scaffold with multistage osteogenic activity, offering a promising strategy for bone defect repair.

## Graphical abstract

**Figure:**
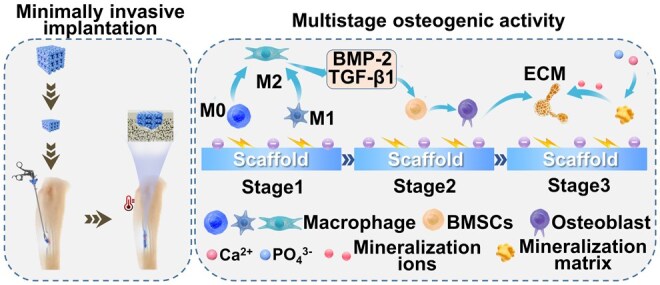

## Introduction

Bone tissue engineering is widely recognized as a potential treatment for large bone defects [1–3]. However, despite decades of development, bone tissue engineering still faces some challenges in clinical translation, making it difficult to achieve widespread application [4–10]. Their poor adaptability for the osteogenic process in current regenerative systems has been considered the main factor. The repair of bone defects involves a series of complex physiological processes [11, 12], which can be divided into early immune microenvironment regulation, stem osteogenic differentiation, and late-stage extracellular matrix mineralization [13–16]. According to the pathological characteristics of different stages of bone repair, choosing suitable bioactive factors, drugs and appropriate stimulation modalities such as ultrasound and electrical signals to adjust the microenvironment has been proven an effective strategy to promote bone regeneration [17]. Ideally, a scaffold with considerable osteogenic effect should have the ability to effectively participate in the three stages of bone repair: early immune microenvironment regulation, stem cell osteogenic differentiation, and late extracellular matrix mineralization, which is called multistage osteogenic activity. However, current bone tissue engineering approaches predominantly address specific stages of osteogenesis rather than the above three bone regeneration stages [18, 19]. Thus, developing bone tissue engineering scaffolds with multistage osteogenic activity would be meaningful for bone tissue engineering.

The bone regeneration scaffolds capable of spatiotemporally delivering drugs and growth factors have shown promise in achieving multistage osteogenic activity [20]. Li *et al.* [21] incorporated hollow mesoporous silica nanoparticles (HMSNs) loaded with alendronate (ALN) and parathyroid (PTH) with calcium magnesium phosphate cement (MCPC) to prepare MCPC/HMSNs@ALN-PTH/GM composite bone cement with dual-drug spatiotemporal sustained-release function. The composite bone cement can realize the spatiotemporal release of ALN, PTH, and bioactive ions to promote vascularization and osseointegration. Coaxial bioprinting is a popular technology for preparing scaffolds with spatiotemporal delivery performance, which can achieve the spatiotemporal release of drugs or growth factors by designing the composition and physicochemical properties of the inner and outer layers of the scaffold fibers. Sun *et al.* [22] fabricated an ion-doped coaxial scaffold using a coaxial printing technology, with hydroxyapatite (HA) as the main component of the scaffold. The inner layer of the scaffold fibers was strontium ion-doped HA (Sr-HA), and the outer layer was silver ion-doped HA (Ag-HA). In the bone repair process, Ag and Sr are released with the degradation of the scaffold in the first and second stages, respectively, which can well meet the requirements of early antibacterial and in the middle and late bone repair treatment. Although the current scaffold with spatiotemporally delivering capacity has made remarkable progress in achieving multistage bone repair, due to the varying physiological conditions in different stages of bone repair, these scaffolds face challenges in the timing and dosage of release as well as the complex design and preparation [23]. As a result, scaffolds with spatiotemporal delivery performance currently used in bone repair can only participate in specific stages of bone repair, predominantly immune microenvironment regulation and stem cell osteogenic differentiation, and have not yet achieved multistage osteogenic activity [24, 25]. Considering that effective participation in multiple stages of bone repair is the key to achieving multistage osteogenic activity, constructing materials with intrinsic properties that can participate in multiple stages of bone repair would significantly advance multistage osteogenic activity. Electroactive materials have been shown to facilitate bone defect repair by modulating the immune microenvironment and demonstrating favorable bone repair effects [26, 27]. Li *et al.* [28] developed a multifunctional bone regeneration membrane (P/T/MXene/ES) by *in situ* doping of MXene 2D nanomaterials with conductive functionality and β-TCP particles into poly(lactic acid-trimethylene carbonate) via electrospinning technology. The composite membrane can promote the osteogenic differentiation of BMSCs and M2 polarization of macrophages through electrical stimulation (ES), demonstrating excellent bone repair effects. Zhao *et al.* [29] fabricated a piezoelectric and aligned nanofiber scaffold via electrospinning technology using zinc oxide (ZnO), poly(ε-caprolactone) (PCL) and poly(vinylidene fluoride) (PVDF) as raw materials. The scaffold can generate electrical signals under ultrasonic stimulation, effectively stimulating the osteogenic differentiation of MC3T3-E1 cells and M2 polarization of macrophages. Additionally, the presence of ZnO allows the scaffold to inhibit local pathogen infection. *In vitro* and *in vivo* experiments demonstrated that the scaffold could effectively inhibit local pathogen infection and promote bone regeneration through the synergistic effect of ZnO nanoparticles and piezoelectric signals. It is known that under physiological conditions, ions such as calcium and phosphate can be attracted by charges and aggregate to form a mineralized matrix [30, 31]. The accumulation of these ions provides the necessary components for extracellular matrix mineralization, thereby accelerating the mineralization process [32, 33]. Although electroactive materials exhibit a promising perspective for constructing scaffolds with multistage osteogenic activity, the investigation of electroactive materials with multistage osteogenic activity in bone repair applications has not been reported yet. Therefore, there is a pressing need to develop electroactive materials with a multistage osteogenic activity that can participate in the multiple bone repair process.

**Figure 1.**
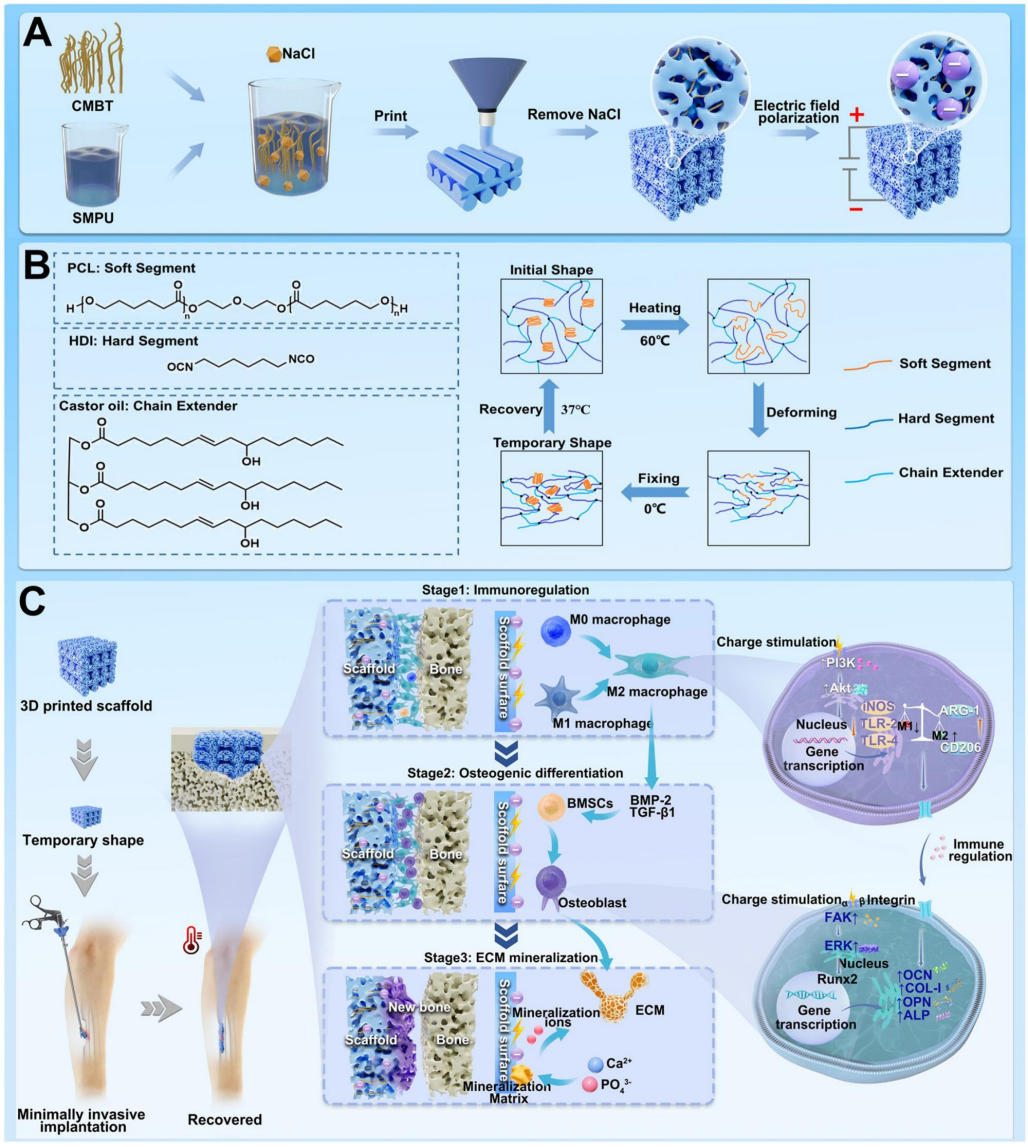
Schematic diagram of the preparation and design concept of the scaffold with multistage osteogenic activity. (**A**) The preparation process of the 3D printed electroactive scaffolds. (**B**) The monomers (one soft segment, one hard segment and one chain extender) are used to prepare SMPUs and the schematic illustration of the mechanism of the shape memory process. (**C**) The mechanism of multistage osteogenic activity. The scaffolds fabricated using 3D printing technology have a customized structure and are capable of structural and functional conversion triggered by physiological temperature, which can be used for minimally invasive implantation. The scaffold carries a large amount of charge after being polarized by a DC electric field. It can regulate the early immune response, osteogenic differentiation of stem cells and extracellular matrix mineralization in late osteogenesis through electrical stimulation, with multistage osteogenic activity.

In this study, a composite scaffold with multistage osteogenic activity composed of shape memory polyurethane (SMPU) and calcium manganese doped barium titanate ceramic short nanofibers (CMBT) was prepared using 3D printed technology (Figure 1). The composite scaffold exhibits dynamic structural and functional transformations in response to physiological temperatures, making it ideal for minimally invasive surgical applications. The composite scaffold can create a surface static voltage of −0.51 kV after being polarized by a direct current (DC) electric field. This charged surface empowers the scaffold to regulate the immune microenvironment by inducing macrophage polarization to the M2 phenotype via activating the PI3K/Akt signaling pathway [34, 35]. This immunomodulatory capability is pivotal for promoting tissue regeneration and healing [36]. Beyond immune modulation, the composite scaffold can also enhance stem cell osteogenic differentiation by activating the FAK/ERK signaling pathway through ES [37, 38]. Furthermore, the charged surface can attract calcium and phosphate ions, which is crucial for extracellular matrix mineralization [39]. By continuously supplying these ions under physiological conditions, the composite scaffold can promote robust extracellular matrix mineralization, accelerating bone repair [33, 40]. Both *in vitro* and *in vivo* evaluations reveal that this composite scaffold orchestrates a synergistic trifecta of actions: regulating the immune microenvironment by promoting macrophage M2 polarization, stimulating stem cell osteogenic differentiation, and driving extracellular matrix mineralization, demonstrating that the composite scaffold has excellent multistage osteogenic activity, which leads to desirable bone defect repair outcomes. In conclusion, we present a novel strategy for preparing 3D printed scaffolds with multistage osteogenic activity, providing a versatile and powerful platform for advancing bone repair technology.

## Materials and methods

### Materials

Barium acetate (Ba(CH_3_COO)_2_), calcium acetate (Ca(CH_3_COO)_2_), manganese acetate (Mn(CH_3_COO)_2_) and tetrabutyl titanate were purchased from Adamas. Polycaprolactone diol (PCL, Mn = 2000), acetylacetone, polyvinyl pyrrolidone (PVP, Mw = 1 300 000), hexamethylene diisocyanate and castor oil were purchased from Aladdin. All other chemicals and solvents were purchased from Macklin.

### Fabrication of CMBT short nanofibers

CMBT nanofibers were fabricated by electrospinning. Briefly, Ba(CH_3_COO)_2_, Ca(CH_3_COO)_2_ and Mn(CH_3_COO)_2_ were dissolved in acetic acid according to a pre-set molar ratio to prepare the electrospinning solution. After all the material was dissolved, tetrabutyl titanate and acetylacetone were added, and the reaction solution was aged at 50°C for 12 h, followed by mixing with a PVP/ethanol solution. The CMBT nanofibers were fabricated through electrospinning (SS-3556H, Yongkang Leye Technology Development, China) and subsequently calcinated at 1000°C for 10 h to obtain CMBT piezoelectric short nanofibers.

### Fabrication of printable ink and scaffold

SMPU prepolymers were prepared by a one-step method. PCL and castor oil were dehydrated under vacuum at 80°C for 12 h. Subsequently, PCL, castor oil and HDI were mixed and stirred at 90°C for 40 min under a nitrogen atmosphere to obtain SMPU prepolymers. CMBT short nanofibers (0, 5, 10, 20, 30 wt%, relative to SMPU prepolymer), sodium chloride (NaCl) particles (250 mesh screen) and SMPU prepolymer were thoroughly blended to prepare printable ink. The printable ink was stored at 4°C. The scaffold was fabricated using a fused deposition molding (FDM) technology (Bio-Architect^®^SR, Regenovo, China), where the scanning rate was 0.001 mm/s, the diameter of the needle was 500 μm and the barrel and nozzle temperatures were 60 and 50°C, respectively. Due to the slow cross-linking of the printable ink at this printing temperature, the viscosity of the ink gradually increases. Therefore, it is necessary to continuously increase the ink extrusion speed during the printing process to ensure the smooth printing of the scaffold. In this study, the ink extrusion speed ranges from 0.001 to 0.1 mm/s, which can ensure the successful printing of the scaffold. After printing, the scaffolds underwent a curing process under a vacuum at 80°C for 12 h and were then immersed in deionized water to remove salt particles. Finally, the composite scaffolds were obtained after vacuum drying.

### Characterization of SMPU and composite scaffold

Fourier transform infrared spectroscopy (FT-IR) was employed to confirm the successful synthesis of SMPU. The sample was placed on the sample table of the FT-IR (NEXUS-670, USA) instrument. The spectral wavelength range is 4000–400 cm^−1^, and the resolution is 0.5 cm^−1^. The microstructure of the CMBT nanofibers and composite scaffolds were observed by scanning electron microscopy (SEM, JSM-7500F, Japan), and the scanning voltage was set at 15 kV. The elemental mapping of characteristic elements (Ca, Mn, Ba and Ti) in the nanofibers was performed under the same parameters as in SEM observation. The lattice structure of the CMBT nanofibers was characterized by X-ray diffraction (XRD, Bruker D8 ADVANCE, China). Differential scanning calorimetry (DSC, DSC2500, USA) determined the scaffold’s shape transition temperature. The shape memory performance and mechanical properties of the scaffolds were assessed using dynamic mechanical analysis (DMA, DMA850, USA) and a mechanical test device. The compress modulus was derived from the stress–strain curves. To evaluate the *in vivo* minimally invasive implantation and shape memory process of the composite scaffold, a male Sprague Dawley rat weighing 170 g and aged three weeks was anesthetized using pentobarbital sodium, and the indocyanine green-loaded temporary shape scaffold was implanted subcutaneously using a minimally invasive procedure. The shape memory behavior of the scaffold under physiological temperature was monitored using an imaging system (IVIS^®^Lumina III, PerkinElmer, USA). The self-adaption irregular defect boundaries performance of the composite scaffold was performed using an irregular bone defect model *in vitro*. The scaffold was heated to 60°C, compressed and fixed at 4°C to fix its temporary shape. After it was implanted into the simulated irregular bone defect model *in vitro*, it was placed in an oven at 37°C to recover its original shape and evaluate its performance of adaptive irregular bone defect boundary. The water contact angle of the scaffold was measured using a contact angle detector (WCA, DSA30, Germany). Degradation assays were conducted in triplicate by incubating the preweighed amount of the scaffolds (1 × 1 × 1 cm^3^) in 10 mL of PBS solutions containing 2000 U/mL lipase (pH = 7.4) under agitation (60 r/min) at 37°C for 7 days. At the end of each time point, scaffolds were removed, washed with deionized water to eliminate residual PBS salts, dried and weighed to quantify the extent of degradation.

### Piezoelectric properties of composite scaffold

Before measuring the piezoelectric properties of the prepared composite scaffold, the scaffold was polarized by a DC electric field of 6 kV/cm at room temperature for 30 min, and the polarized scaffold was named *p*3DES and *p*3DS, respectively. Then, the surface electrostatic voltage, surface zeta potential and piezoelectric coefficient (d_33_) of the scaffolds were measured by the surface electrostatic voltage detector (JH-TEST, China), solid zeta potential detector (Anton Paar surpass3, Austria) and the quasistatic d_33_ m device (ZJ-3A, China) at room temperature. The scaffolds were immersed in the medium and removed every 5 h, washed with deionized water and dried under vacuum. Then, the surface electrostatic voltage stability of the polarized scaffolds was measured by an electrostatic voltage detector. The cell growth medium was composed of α-Modified Eagle Medium (α-MEM, Cyagen Bioscience Inc, Guangzhou, China) supplemented with 10% (v/v) fetal bovine serum (FBS, Ausbian, Australia) and 1% (v/v) penicillin/streptomycin (Gibco, USA).

### *In vitro* biocompatibility of composite scaffold

BMSCs were extracted from the femoral bone marrow of 2-week-old SD rats provided by the Shanghai Laboratory Animal Center (SLAC). The cells were cultured in a growth medium, and the culture chamber environment was maintained at 37°C, saturated humidity and 5% CO_2_. Cells from passages 3–5 were used for subsequent *in vitro* experiments, with the culture medium being refreshed every other day. Before cell experiments, the materials underwent an 8-h ultraviolet sterilization treatment.

BMSCs were seeded in a 96-well cell (1 × 10^4^ cells/well) culture plate containing sterilized scaffolds (*d* = 6 mm) for cell proliferation experiments. After 1, 4 and 7 days of cell culture, the culture medium was removed, and cell counting kit-8 (CCK-8, Beyotime, China) solution (10% in DMEM/F-12) was added to each well to incubate the cells. After a 30-min incubation, the CCK-8 solution was collected, and the absorbance at 405 nm was measured using a microplate reader (Multiskan GO, USA) to assess cell proliferation. The scaffolds’ biocompatibility and cell migration ability were assessed using live/dead fluorescence staining, hemolysis characterization, cell morphology and scratch assays. For live/dead fluorescence staining, BMSCs (4 × 10^4^ cells/well) were seeded on sterilized scaffolds (*d* = 12 mm) for 1, 4 and 7 days. After removing the culture medium, the cells were stained with a calcein-AM/PI double staining kit, and observation was done using a fluorescence microscope (IS100, Japan). For hemolysis characterization, the composite scaffold (10 mg) was immersed in a centrifuge tube containing 2 ml of 4% red blood cell suspension and incubated at 37°C for 1 h. Subsequently, hemolysis was observed and photographed, and finally, quantitative analysis was performed using a microplate reader. For cell morphology, cells (4 × 10^4^ cells/well) were seeded on sterilized scaffolds (*d* = 12 mm) for 1 and 7 days. After removing the culture medium, fixation was performed with 4% paraformaldehyde, followed by staining with DAPI and phalloidin. Cell morphology, including cell skeleton and nuclear morphology, was observed using a fluorescence microscope. For scratch assays, cells (4 × 10^4^ cells/well) were cultured on sterilized scaffolds (*d* = 12 mm) for 24 h, followed by scratch treatment. Live/dead fluorescence staining was performed on 0, 24 and 48 h after the scratch treatment to observe cell migration ability.

### *In vitro* macrophage immune regulation

A murine-derived macrophage cell line RAW264.7 was used for inflammation response assessment. After seeding the cells on scaffolds (*d* = 12 mm) for 2 days, the cells were fixed with 4% paraformaldehyde, then permeabilized with 0.1% Triton X-100 and blocked with 2% BSA. After blocking, the cells were incubated overnight at 4°C with monoclonal antibodies against CD86 and CD206. Subsequently, the cells were incubated with secondary antibodies at room temperature for 1 h. The cell nuclei were then stained with DAPI at room temperature for 5 min, and observation was conducted using fluorescence microscopy.

Flow cytometry was used to identify M1 and M2 phenotypes of macrophages. After seeding the cells on the scaffolds for 2 days, the cells were detached, centrifuged and washed three times with PBS. The cells were resuspended in 1 mL of PBS, stained with CD86 and CD206 dyes and incubated on ice in the dark for 1 h. After centrifugation, the cells were resuspended in PBS for flow cytometry analysis.

The expression of CD86 and CD206 proteins in macrophages was analysed using western blotting. The cells seeded on the scaffolds were lysed using RIPA cell lysis buffer, and the protein concentration was determined using a BCA protein concentration assay kit. Subsequently, sodium dodecyl sulfate-polyacrylamide gel electrophoresis (SDS-PAGE) was conducted. After electrophoresis, proteins were transferred onto a PVDF membrane. The membrane was then blocked with TBST blocking solution containing 5% skimmed milk for 1 h. After blocking, it was incubated overnight at 4°C with primary antibodies against CD86 and CD206. Following washes with TBST three times, the membrane was incubated with secondary antibodies at room temperature for 1 h. Finally, chemiluminescence imaging was performed using a chemiluminescent detection system.

The expression of genes associated with the M1 (iNOS, TLR-2, TLR-4) and M2 (ARG-1, CD206) phenotypes in macrophages was analysed using RT-qPCR. After seeding the cells on the scaffolds for 4 and 7 days, the RNA was extracted with a Trizol RNA extraction reagent. The concentration of extracted RNA was determined at 260 nm using a nanospectrophotometer. The extracted RNA was then reverse-transcribed into cDNA using a reverse transcription kit. Finally, SYBR Green reagent was added, and RT-qPCR was conducted using the ABI PRISM 7500 real-time PCR system.

Additionally, the secretion levels of osteogenic factor (TGF-β1 and BMP-2) in macrophages were evaluated through immunofluorescence staining, and the target proteins (TGF-β1 and BMP-2) in the supernatant were tested using western blotting. The regulatory role of M2 macrophage in the osteogenic differentiation process was verified through ALP and ARS staining. RAW264.7 cells were co-cultured with composite scaffolds for 4 days and then collected the medium to culture the BMSCs for alkaline phosphatase (ALP) and alizarin red (ARS) staining.

### Macrophage polarization-related signaling pathways

The expression of intracellular PI3K, P-PI3K, Akt and P-Akt proteins was evaluated using western blotting. After seeding cells (1.6 × 10^5^ cells/well) on the scaffolds (*d* = 24 mm) for 2 days, the cells were lysed using RIPA cell lysis, and the protein concentration was determined using a BCA protein concentration assay kit. Subsequently, SDS-PAGE was conducted. After electrophoresis, proteins were transferred onto a PVDF membrane. The membrane was then blocked with TBST blocking solution containing 5% skimmed milk for 1 h. After blocking, it was incubated overnight at 4°C with primary antibodies. Following washes with TBST three times, the membrane was incubated with secondary antibodies at room temperature for 1 h. Finally, chemiluminescence imaging was performed using a chemiluminescent detection system.

### Biomineralization of composite scaffold

To assess the biomineralization performance of composite scaffolds, various polarized scaffolds (1 × 1 cm^2^) were submerged in 1.5-fold SBF (1.5 × SBF, 10 mL) for 7 days at 37°C, the 1.5 × SBF contained Na^+^ 213 mM, K^+^ 7.5 mM, Mg^2+^ 2.25 mM, Ca^2+^ 3.75 mM, Cl^−^ 221.7 mM, ${\text{HCO}}_{3}^{-}$ 6.3 mM, ${\text{HPO}}_{4}^{2-}$ 1.5 mM and ${\text{SO}}_{4}^{2-}$ 0.75 mM. The pH of SBF was adjusted to 8.5 by Tris-HCL, and the SBF solution was refreshed every day. After the immersion period, the scaffolds were retrieved, washed with deionized water and dried at room temperature. Finally, the morphological and elemental analyses of surface mineralized nodules were performed using SEM and mapping.

### *In vitro* osteogenic differentiation of composite scaffold

To assess the *in vitro* osteogenic differentiation effect of the composite scaffolds, ALP and ARS staining were conducted. For ALP staining, BMSCs (5 × 10^4^ cells/well) were seeded on sterilized scaffolds (*d* = 12 mm) and cultured for 24 h. The medium was then replaced with an osteogenic induction medium, which was refreshed every two days. After 7 and 14 days of cell culture, the culture medium was removed, and the cells were washed with PBS three times. Fixation was carried out with 4% paraformaldehyde for 30 min, washed with PBS three times and then prepared ALP staining solution was added, and the cells were stained at room temperature for 30 min. Finally, the staining solution was removed, and after three PBS washes, observation and photography were conducted using a fluorescence microscope. Quantitative analysis of alkaline phosphatase secretion by cells was conducted using an alkaline phosphatase assay kit. For ARS staining, after 14 and 21 days of cell culture, the medium was removed, and cells were fixed with 4% paraformaldehyde for 30 min. Subsequently, ARS staining solution was then added, and the cells were stained at 37°C for 30 min. After staining, the solution was removed, and the cells were washed three times with PBS. Observed and photography were then conducted using a fluorescence microscope. To analyse mineralization levels, the culture medium was removed after ARS staining, and a 10% hexadecylpyridinium chloride solution was added to each well. The cells were then incubated at room temperature for 1 h, and absorbance was subsequently measured at 562 nm using a microplate reader.

Quantitative analysis of the expression of osteogenic-related genes [ALP, osteopontin (OPN), osteocalcin (OCN) and collagen I (COL-I)] was conducted using real-time fluorescence quantitative polymerase chain reaction (RT-qPCR) (Table 1). The culture medium was removed after inducing BMSCs (1.6 × 10^5^ cells/well) culture in a six-well plate for 7 and 14 days. Subsequently, 1 mL of Trizol RNA extraction reagent was added to each well to extract RNA. The concentration of extracted RNA was determined at 260 nm using a nanospectrophotometer. Then, the extracted RNA was reverse-transcribed into cDNA using a reverse transcription kit. Finally, SYBR Green reagent was added, and RT-qPCR was conducted using the ABI PRISM 7500 real-time PCR system.

**Table 1.** Primers designed for genes related to osteogenic differentiation of BMSCs and immune response of RAW264.7 cells

Name	Gene forward (5′–3′)	Reverse (3′–5′)	Size
GAPDH	CTGGAGAAACCTGCCAAGTATG	GGTGGAAGAATGGGAGTTGCT	43
ALP	GACAAGAAGCCCTTCACAGC	ACTGGGCCTGGTAGTTGTTG	40
OPN	GATGAACAGTATCCCGATGCCA	GTCTTCCCGTTGCTGTCCTGA	43
OCN	CAACCCCAATTGTGACGAGC	GGCAACACATGCCCTAAACG	40
COL-1	TCAAGATGGTGGCCGTTACT	TCTTTGCATAGCACGCCATCG	41
CD206	TCAATGCCACTGCCATGCCTAC	AGCTTGCCGTGCGTCTTGC	43
ARG-1	CAGCAAAGCAGACAGAACTAAG	AGAAAGGAACTGCTGGGATAC	41
iNOS	GAGACAGGGAAGTCTGAAGCAC	CCAGCAGTAGTTGCTCCTCTTC	44
TLR-2	ACAGCAAGGTCTTCCTGGTTCC	GCTCCCTTACAGGCTGAGTTCT	44
TLR-4	AGCTTCTCCAATTTTTCAGAACTTC	TGAGAGGTGGTGTAAGCCATGC	47
Integrin α1	CCTGTACTGTACCCAATTGGATGG	GTGCTCTTATGAAAGTCGGTTTCC	48
Integrin α2	CACAGTTCATTTTTAGGTTACT	CACATTGCCATGCTTGTTAACA	44
Integrin α5	ACAGTTCGAGCCCATGGCT	CTGAACACATTCTTTATGCTC	40
Integrin β1	CTACTGGTCCCGACATCATCC	TGACCACAGTTGTCACGGCAC	42

The expression of bone-related proteins was analysed using western blotting. Cells were lysed with RIPA cell lysis buffer, and the protein concentration was determined using a BCA protein assay kit. Subsequently, SDS-PAGE was conducted. After electrophoresis, proteins were transferred onto a PVDF membrane. The membrane was then blocked with TBST blocking solution containing 5% skimmed milk for 1 h. After blocking, it was incubated overnight at 4°C with primary antibodies against ALP, OPN, OCN and COL-I. Following washes with TBST three times, the membrane was incubated with secondary antibodies at room temperature for 1 h. Finally, chemiluminescence imaging was performed using a chemiluminescent detection system.

### Osteogenic-related signaling pathways

RT-qPCR was employed to analyse the expression of integrin genes in BMSCs. After seeding BMSCs (1.6 × 10^5^ cells/well) on the scaffolds (*d* = 24 mm) for 6 h, genes were extracted using a Trizol RNA extraction reagent. The expression of integrin α1, α2, α5 and β1 was assessed by RT-qPCR, with GAPDH as the reference gene.

The expression of intracellular FAK, P-FAK, ERK and P-ERK proteins was evaluated by western blotting. After seeding cells (1.6 × 10^5^ cells/well) on the scaffolds (*d* = 24 mm) for 2 days, the cells were lysed using RIPA cell lysis, and the protein concentration was determined using a BCA protein concentration assay kit. Subsequently, SDS-PAGE was conducted. After electrophoresis, proteins were transferred onto a PVDF membrane. The membrane was then blocked with TBST blocking solution containing 5% skimmed milk for 1 h. After blocking, it was incubated overnight at 4°C with primary antibodies. Following washes with TBST three times, the membrane was incubated with secondary antibodies at room temperature for 1 h. Finally, chemiluminescence imaging was performed using a chemiluminescent detection system.

### *In vivo* bone defect repair efficacy

All the experimental animal procedures were performed by following local animal welfare laws and guidelines and approved by the Animal Ethics Committee of Donghua University (No: DHUEC-NSFC-2022-27, Date: March 1, 2022). Thirty male Sprague Dawley rats (7 weeks old, 350 g) were randomly divided into five groups: *p*3DES (implanted with polarized 3DES scaffolds), 3DES (implanted with unpolarized 3DES scaffolds), *p*3DS (implanted with polarized 3DS scaffolds), 3DS (implanted with unpolarized 3DS scaffolds), and control group (without implanted). Rats were anesthetized using chloral hydrate (0.2 ml, 50 g), and then skull defects were created on both sides of the sagittal suture in the rat’s head by drilling two circular defects (*d* = 5 mm). Scaffolds were implanted into the defect sites after ethylene oxide sterilization. We set four samples at each time point; the tissues were sutured after the scaffolds were implanted.

After 4 and 8 weeks of implanting, euthanize the rats and use micro-computed tomography (micro-CT) (Quantum FX, PerkinElmer, USA) scans to image the calvarial defects with surrounding normal bone. The CTAn (SkyScan software) was used to analyse the data obtained from micro-CT scanning. Subsequently, the specimens were stored in a 4% neutral formalin buffer solution for two days. The cranial defects with scaffolds were decalcified in 10% EDTA/HCl for 30 days. Following decalcification, it underwent ethanol gradient dehydration and was embedded in paraffin. The specimen was sectioned into 15 mm thick cross-sections for subsequent staining processes such as Hematoxylin and Eosin (H&E) and Masson’s trichrome staining.

Pro-inflammatory and anti-inflammatory analyses were conducted using immunohistochemistry and immunofluorescence detection. For immunohistochemical staining, deparaffinized tissue sections were incubated with primary antibodies against iNOS (M1 macrophage marker, Affinity Biosciences, USA) and CD206 (M2 macrophage marker, Affinity Biosciences, USA). Images were captured using a Nanozoomer digital slide scanner (Hamamatsu, Japan). Deparaffinized tissue sections were treated with primary antibodies against iNOS and CD206 (Affinity Biosciences, USA) for immunofluorescence staining, with cell nuclei stained using DAPI. Images were acquired using confocal fluorescence microscopy.

### Statistical analysis

Quantitative data were analysed using one-way analysis of variance (ANOVA) in Origin 2024. All quantitative data are expressed as the mean ± standard deviation for *n* ≥ 3. *P* values ≤0.05 indicate a statistical difference between the groups. In contrast, *P* values ≤0.01 indicate a statistically significant difference between the groups.

## Results

### Preparation and characterization of CMBT nanofibers

**Figure 2.**
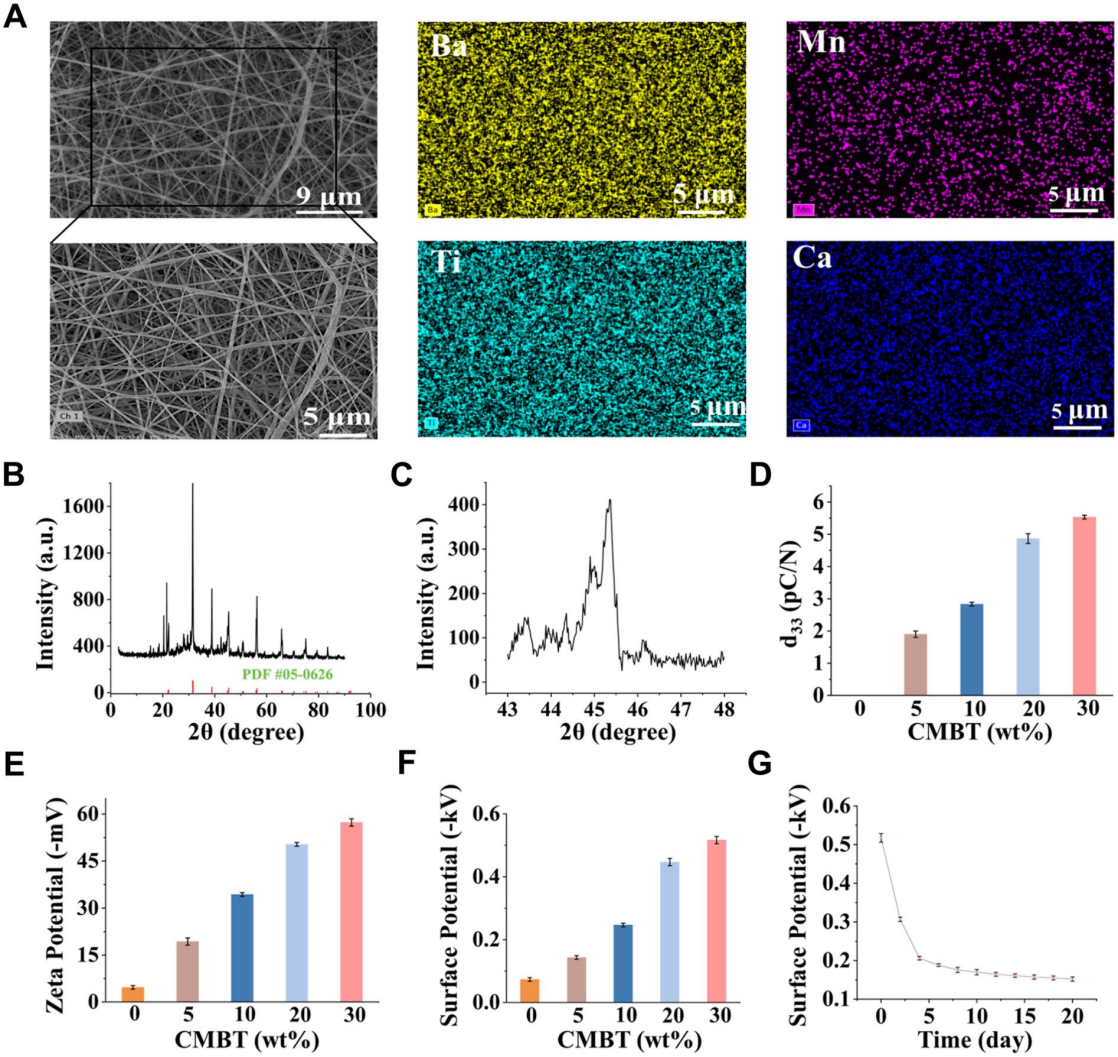
Characterization of CMBT nanofibers and scaffolds. (**A**) SEM image and mapping image of CMBT nanofibers. (**B** and **C**) The XRD pattern of CMBT nanofibers and the characteristic peak of the tetragonal phase are located at 45° in the XRD pattern of CMBT nanofibers. (**D**) Piezoelectric coefficient (*d*_33_) of composite scaffolds with different CMBT short nanofiber content. (**E**) Zeta potential of composite scaffolds with different CMBT short nanofiber content. (**F**) Surface static voltage of scaffolds with different CMBT short nanofiber content. (**G**) Surface static voltage stability of scaffolds with 30 wt% CMBT short nanofibers (*n* = 3; error bars represent standard deviation).

Firstly, CMBT nanofibers were prepared as the piezoelectric component using the electrospinning technology [41, 42]. The morphology and elemental distribution of the nanofibers were characterized through SEM and elemental mapping. As shown in Figure 2A, the average diameter of the nanofibers was 160 ± 14 nm, and elemental mapping images indicated that calcium, manganese, titanium and barium elements were evenly distributed in the nanofibers, confirming the successful synthesis of CMBT nanofibers. The presence of calcium, manganese, titanium and barium elements in nanofibers was further analysed using X-ray photoelectron spectroscopy (XPS). As shown in Supplementary Figure S1, according to XPS peak-differentiating and -imitating results, revealing binding energies of 458.5 and 463.9 eV for Ti 2p, 530 eV and 531.7 eV for O 1 s, 794.9 eV for Ba 3d, 346.8 eV for Ca 2p and 641.4 eV for Mn 2p, respectively. The lattice structure of nanofibers was characterized by XRD, and the results (Figure 2B and C) indicated that the obtained XRD spectrum was consistent with the diffraction peaks of barium titanate. The splitting peak at 2*θ* = 45° was the characteristic peak of tetragonal perovskites. The piezoelectric properties of barium titanate are derived from the tetragonal phase, further demonstrating the successful preparation of CMBT piezoelectric nanofibers.

### Preparation and characterization of printable ink and composite scaffold

Polyurethane was selected as the shape memory material in the composite scaffold. As a traditional shape memory polymer, polyurethane has been proven to possess excellent shape memory performance, allowing for minimally invasive implantation and adaptation to irregular bone defect boundaries [43, 44]. Moreover, the mechanical properties and shape memory activation temperature of polyurethane can be flexibly adjusted according to needs by modifying the ratio of the soft and hard segments [45]. Therefore, composite scaffolds prepared from polyurethane have excellent shape memory performance, tunable mechanical properties and shape memory activation temperature, enabling minimally invasive implantation and adaptive to irregular bone defect boundaries. The SMPU was synthesized using PCL, HDI and castor oil as raw materials using a one-step method. In this formulation, PCL is the soft segment, providing flexibility and toughness. HDI acts as the hard segment, offering hardness and wear resistance. Castor oil plays a role in plasticization and toughening (Supplementary Figure S2). The FT-IR result of SMPU is shown in Supplementary Figure S3. The peaks at 3390 and 1722 cm^−1^ corresponded to –N–H and C=O stretching vibrations, respectively, indicating the formation of an amido bond. The characteristic peak (2250–2270 cm^−1^) for the NCO– group is not found in the spectrum, indicating that the NCO– group reacts completely. The above results proved the successful synthesis of SMPU. The melting temperature (*T_m_*, regarded as shape memory temperature) of SMPU was assessed using DSC, and the results are shown in Supplementary Figure S4. By varying the molar ratio of PCL to castor oil, the *T_m_* of SMPU can be tuned. It was observed that decreasing the molar ratio of PCL led to an increase in the *T_m_* of SMPU. The reduction of PCL content is accompanied by the increase of castor oil content, which increases the length of the molecular chain and the number of cross-link points in SMPU and improves the heat resistance of SMPU. To achieve a better physiological temperature responsiveness of the SMPU, a molar ratio of 11:9 (*T_m_* = 27°C) was selected for subsequent scaffold preparation. The degradation behavior of SMPU was investigated by immersing SMPU in PBS containing 2000 U/ml lipase at 37°C. The degradation kinetics of SMPU is shown in Supplementary Figure S5. The results showed that the degradation rate of SMPU could reach 9.6% within 7 days, proving that it has excellent degradation characteristics and is suitable for bone defect repair. Subsequently, the SMPU prepolymer was mixed with NaCl particles with average sizes ranging from 60 to 80 µm to prepare the printable ink. To obtain the appropriate proportion of ink preparation, the cylinders with a diameter of 1 cm and a height of 2 cm were prepared for shape retention experiments. Considering the curing temperature of polyurethane, which ranges from 60 to 80°C [43], the shape retention experiments were conducted at 80°C. The results (Supplementary Figure S6) indicated that the optimal shaping retention effect for the printable ink was achieved when the mass ratio of SMPU to NaCl was equal to or exceeded 1:2.5. Additionally, although the shape retention performance of the printable ink with a mass ratio of 1:3 was better than that of the printable ink with a mass ratio of 1:2.5, we found that the ink with this mass ratio was difficult to print smoothly in the subsequent printing process of the scaffold, which was manifested as difficult extrusion and the extruded filament was easy to break. Thus, the printable ink with a mass ratio of 1:2.5 was selected for the subsequent scaffold printing. The scaffold was printed using FDM technology, and the printed scaffold was cured at 80°C. The cured scaffold was then immersed in deionized water for 48 h to remove the NaCl particles. To ensure the complete removal of NaCl particles, the water was replaced several times and combined with a gentle stirring process (200 rpm) to facilitate the leaching process. After the removal of the NaCl particles, SEM was used to observe the scaffold to confirm the complete removal of NaCl particles (Supplementary Figure S7). The result showed that no NaCl particles remained in the scaffold, and the scaffold maintained its structural integrity, confirming the successful preparation of the scaffold.

CMBT short nanofibers were fabricated by grinding the CMBT nanofibers and further uniformly mixed with printable ink in varying mass ratios (5, 10, 20 and 30 wt%) to prepare CMBT/SMPU printable ink. The CMBT/SMPU composite scaffold was prepared using FDM technology. CMBT short nanofibers were uniformly dispersed in the composite scaffold without aggregation (Supplementary Figure S8), confirming the successful preparation of the composite scaffold. The degradation behavior of the CMBT/SMPU composite scaffold with different CMBT short nanofiber contents (5, 10, 20 and 30 wt%) was investigated under the same conditions as the degradation experimentation of SMPU. The degradation kinetics of CMBT/SMPU composite scaffolds are shown in Supplementary Figure S9. The results showed that the degradation rates of each experimental group ranged from 9.4% to 9.9% within 7 days, indicating that the addition of CMBT short nanofibers had no significant effect on the degradation behavior of SMPU.

Subsequently, the piezoelectric properties of the fabricated composite scaffolds were evaluated. Before assessing the piezoelectric properties, all the prepared composite scaffolds were subjected to DC electric field polarization. The d_33_ is a vital parameter to describe the piezoelectric properties of piezoelectric materials, which represents the proportional relationship between the charge generated by the piezoelectric strain along a particular direction and the applied electric field under the action of the electric field [46, 47]. The zeta potential represents the tendency and potential energy difference of the charge distribution on the surface of the material [48]. The results indicated that increasing CMBT short nanofiber content progressively enhanced the composite scaffold’s d_33_ (Figure 2D) and zeta potential (Figure 2E). When the CMBT short nanofiber content reached 30 wt%, the zeta potential and d_33_ values were −57.3 mV and 5.5 pC/N, respectively, which are close to the physiological potential (−60 to −100 mV) and piezoelectricity (0.7–2.3 pC/N) of human bone [49]. Subsequently, the surface static voltage of the composite scaffold was measured, and the results are shown in Figure 2F. With an increase in CMBT short nanofiber content, the surface static voltage of the composite scaffold also increased. However, the printability of the printable ink decreased when the CMBT short nanofiber content exceeded 30 wt%. Therefore, in conjunction with the result of piezoelectric performance characterization, the composite scaffold with a CMBT short nanofiber content of 30 wt% was selected for subsequent experiments and named 3D printed electroactive scaffolds (3DES) (the scaffold without CMBT short nanofibers was named 3DS). Due to the presence of CMBT short nanofibers, the scaffold can generate a surface static voltage when subjected to pressure [50]. Therefore, the piezoelectric properties of the composite scaffold were further evaluated by assessing the surface static voltage of the composite scaffold under different pressures. The result is shown in Supplementary Figure S10; the result confirmed that as the applied pressure increased, the surface static voltage of the composite scaffold also increased, demonstrating the excellent piezoelectric properties of the composite scaffold. To achieve multistage osteogenic activity of the scaffold, the static voltage generated on the scaffold’s surface must remain stable for an extended period under physiological conditions. Therefore, the surface static voltage stability of the composite scaffold was evaluated (Figure 2G). The result showed that the surface static voltage of the composite scaffold exhibited a biphasic pattern, characterized by a rapid decrease during the first 6 days, followed by a gradual reduction in the rate of decline, stabilizing at −0.15 kV on the 20th day. Under stable conditions, the surface static voltage of the composite scaffold is sufficient to stimulate osteogenic differentiation of stem cells [51]. These findings confirm that the prepared composite scaffold possesses excellent piezoelectric properties. The charge carried on the surface of the polarized composite scaffold can exist stably for a long time and could meet the needs of multistage osteogenesis.

### Shape memory performance of composite scaffold

**Figure 3.**
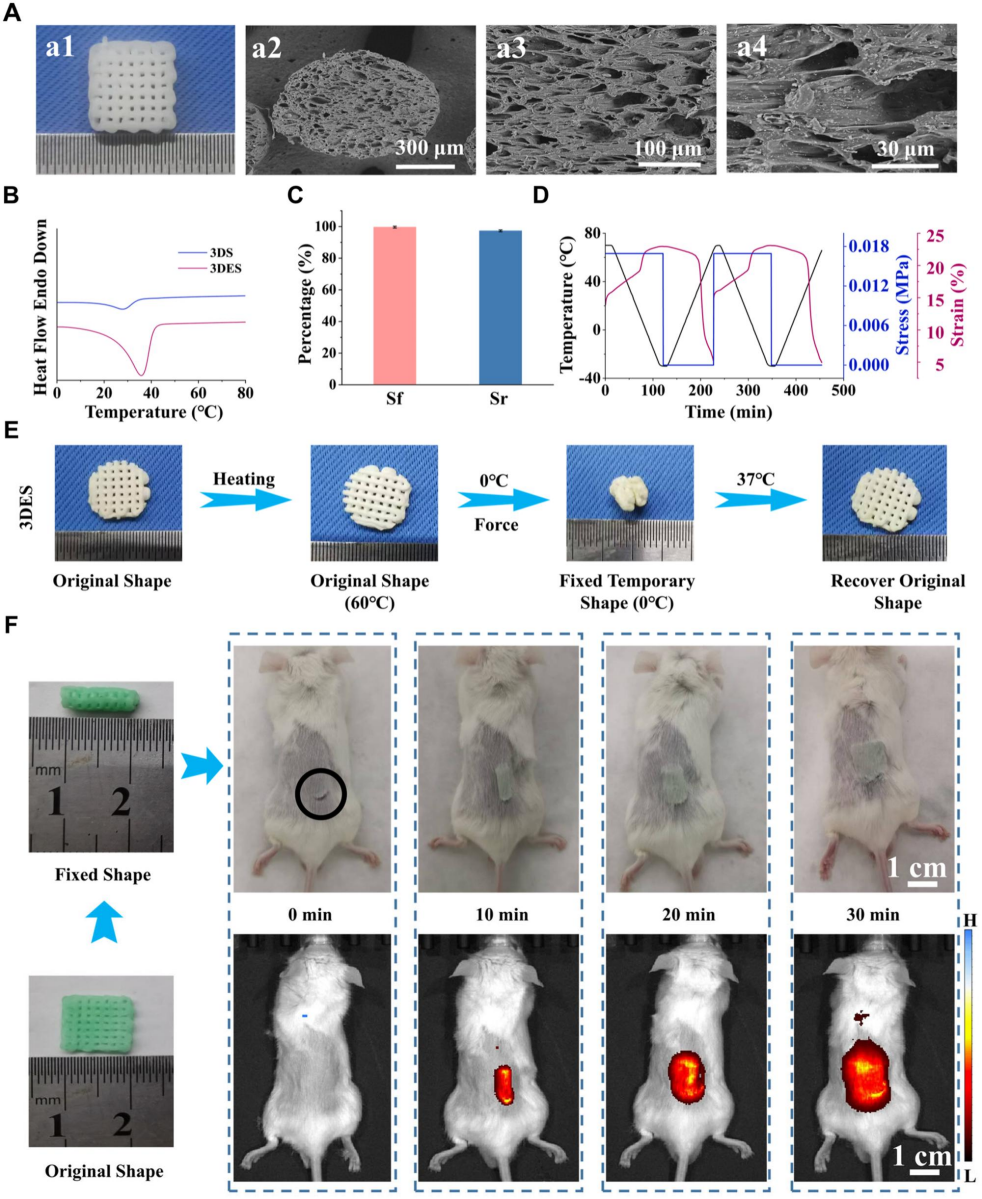
The shape memory property of scaffolds. (**A**) The overall image (a1) and SEM image at different magnifications (a2–a4) of 3DES. (**B**) DSC curve of 3DES and 3DS. (**C**) Shape fixed rate (Sf) and shape recovery rate (Sr) of 3DES. (**D**) Shape memory cycle of 3DES. (**E**) *In vitro* shape memory behavior of 3DES. (**F**) *In vivo* simulation of 3DES for the minimally invasive implantation and shape memory process (*n* = 3; error bars represent standard deviation).

After high-temperature curing (80°C) and removal of the NaCl, the composite scaffold maintained its original printed structure and exhibited porous microstructure. The SEM images of 3DES are shown in Figure 3A. The CMBT short nanofibers were uniformly dispersed in the 3DES without aggregation, indicating the successful preparation of the composite scaffold. Supplementary Figures S10B and S11A show the composite scaffold’s mechanical properties. The mechanical property was assessed at physiological temperature because the scaffold will be used for *in vivo* bone defect repair. The temperature was set to 37°C to simulate physiological temperature. The results showed that the compressive modulus of 3DS is 2.3 ± 0.2 MPa, and the compressive modulus of the 3DES is 4.6 ± 0.5 MPa. The addition of CMBT short nanofibers enables the electroactivity of 3DES and serves as nanofillers to improve mechanical properties. According to the literature, this level of mechanical property can meet the need for bone defect repair [4].

Supplementary Figure S12 shows the water contact angle results of the composite scaffolds. Compared to the non-polarized 3DES, the water contact angle of the polarized 3DES decreased from 90.0° to 68.5°. After polarization, the charges carried on the surface of the composite scaffold can bind water molecules, thereby enhancing the scaffolds’ hydrophilicity [52]. It is known that a water contact angle between 60° and 80° is favorable for cell adhesion and proliferation [53], indicating that polarized 3DES exhibits excellent hydrophilicity, which is conducive to cell adhesion. The *T_m_* of composite scaffolds was measured using DSC. As shown in Figure 3B, the *T_m_* of the 3DS was 27°C, while the *T_m_* of the 3DES was 37°C. Because the addition of CMBT short nanofibers hinders the molecular motion of SMPU, the 3DES requires a higher temperature to trigger shape memory behavior. These findings indicate that physiological temperature can trigger both scaffolds’ shape memory behavior. Figure 3C and D and Supplementary Figure S13 are the quantitative analysis of the shape memory performance of 3DS and 3DES. The results showed that the addition of CMBT short nanofibers had no significant effect on the shape memory performance of the SMPU scaffold, and the Sf and Sr of both scaffolds exceeded 97%. The qualitative analysis of the composite scaffold’s shape memory performance is shown in Figure 3E. The scaffold was programmed into a temporary shape at 60°C, fixed the temporary shape at 0°C and recovered the original shape at 37°C. The results showed that the Sf of 3DES exceeded 98%, and the Sr of 3DES at 37°C was above 99%. When the temperature reaches above *T_m_*, the soft segments in the SMPU molecular chain transition from a crystalline state to a molten state, then the SMPU can deform and be prepared into various shapes under external forces. When the temperature drops to 0°C, the soft segments will crystallize again, allowing the SMPU to retain a fixed temporary shape. When the temperature rises above the *T_m_* again, the molecular chains transition from a crystalline state to a molten state again, and the presence of hard segments can prevent plastic deformation during the deformation process, resulting in a higher shape recovery rate of the SMPU. The minimally invasive implantation and self-adaption irregular defect boundaries process of 3DES are shown in Supplementary Figure S14. After minimally invasive implantation of the scaffold into the simulated defect site, the scaffold can completely restore the original shape and adapt to irregular bone defect boundaries, demonstrating the potential clinical significance of the scaffold.

To confirm the composite scaffold’s *in vivo* minimally invasive implantation performance and physiological temperature-triggered shape recovery performance, we implanted a fixed shape composite scaffold loaded with the fluorescent dye indocyanine green subcutaneously in a rat via a minimally invasive method. Fluorescence imaging was used to assess the composite scaffold’s shape recovery performance under physiological temperature, and the results are shown in Figure 3F. After the composite scaffold was implanted, it was able to fully recover its original shape within 30 min under physiological temperature, indicating that the composite scaffold can achieve shape recovery triggered by physiological temperature, demonstrating excellent shape memory performance.

### *In vitro* biocompatibility assessment of composite scaffold

**Figure 4.**
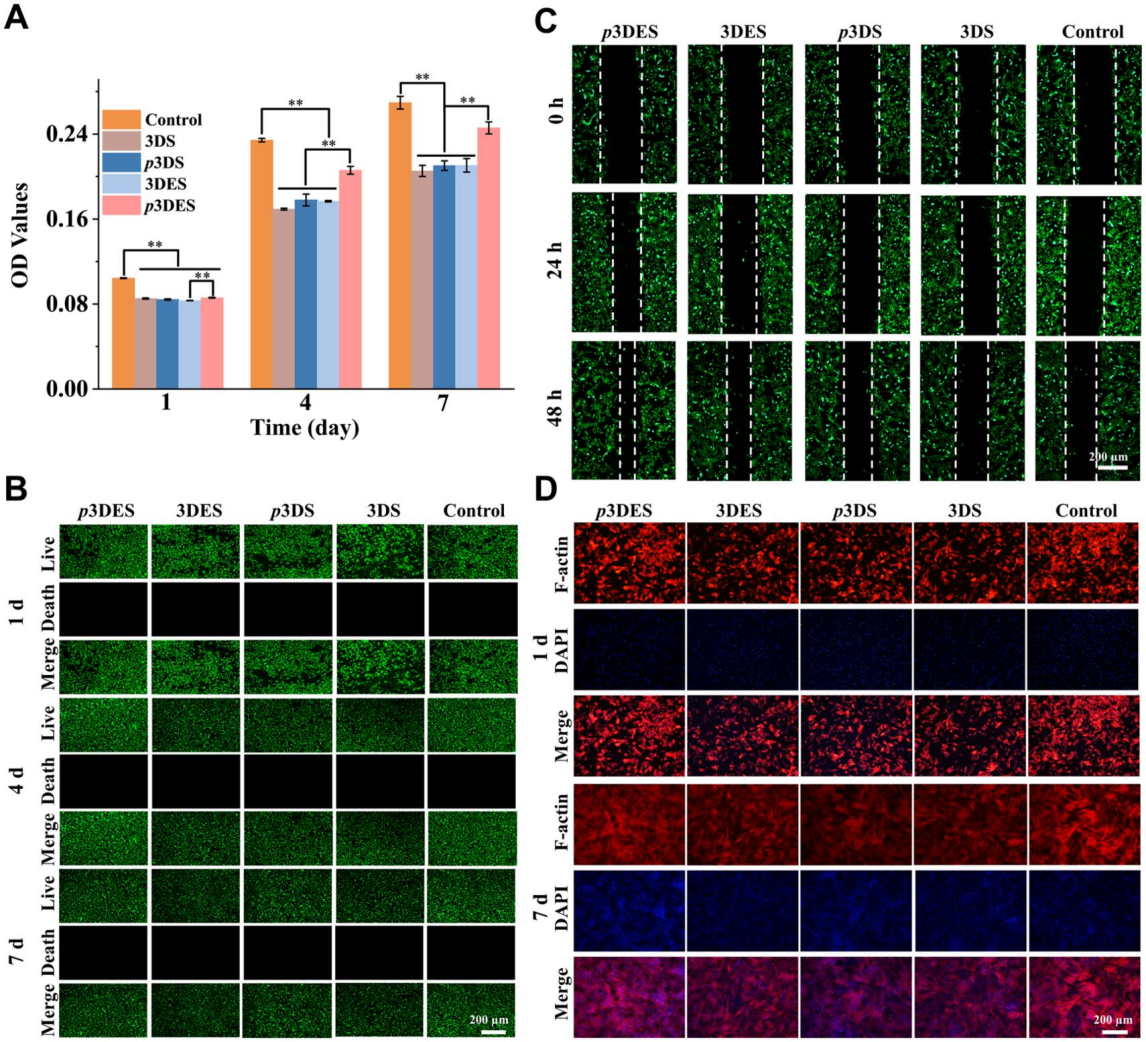
*In vitro* biocompatibility assessment of scaffolds. (**A**) Proliferation of BMSCs treated with different scaffolds for 1, 4 and 7 days. (**B**) Live/dead fluorescent staining of BMSCs treated with different scaffolds for 1, 4 and 7 days. (**C**) Cell scratch assay results after incubation for 0, 24 and 48 h with different scaffolds. (**D**) Immunofluorescence staining of BMSCs with phalloidin and DAPI treated with different scaffolds for 1 and 7 days. The control group was operated without scaffold treatment. **P < 0.01, highly significant (*n* = 3; error bars represent standard deviation).

BMSCs were used to evaluate the biocompatibility of the composite scaffold. The CCK-8 results are shown in Figure 4A, indicating that BMSCs from all experimental groups exhibited sustained proliferation. Notably, BMSCs treated with polarized 3DES (*p*3DES) exhibited the highest proliferation rate compared to 3DES, *p*3DS and 3DS, indicating that polarized 3DES significantly enhances the proliferation of BMSCs. Figure 4B shows the live/dead fluorescence staining results. The green and red fluorescence represent live and dead cells, respectively. The results demonstrated that BMSCs in all experimental groups survived normally, and cells treated with polarized 3DES exhibited robust green fluorescence, indicating a higher proportion of live cells, consistent with previous studies that ES can promote stem cell adhesion and proliferation [54].

The hemolysis test further proved the biocompatibility of the composite scaffold. According to the results (Supplementary Figure S15), the hemolysis rates of all the groups were lower than 0.4, while complete hemolysis occurred in the positive control group (deionized water), which proved the excellent biocompatibility of the composite scaffold. The cell scratch results in Figure 4C indicated that the polarized 3DES can effectively promote cell migration and diffusion, consistent with the previous studies that ES can stimulate cell migration and diffusion [54]. The cell skeleton and nucleus staining results are shown in Figure 4D. The results revealed that the morphology of cell skeletons remained normal in all experimental groups, suggesting that the scaffold treatment did not adversely affect cell morphology, adhesion and diffusion. These results demonstrate that the charge carried on the surface of the composite scaffold after electric field polarization could promote cell adhesion and proliferation, demonstrating the excellent biocompatibility of the composite scaffolds.

### *In vitro* early immune microenvironment regulation of composite scaffold

**Figure 5.**
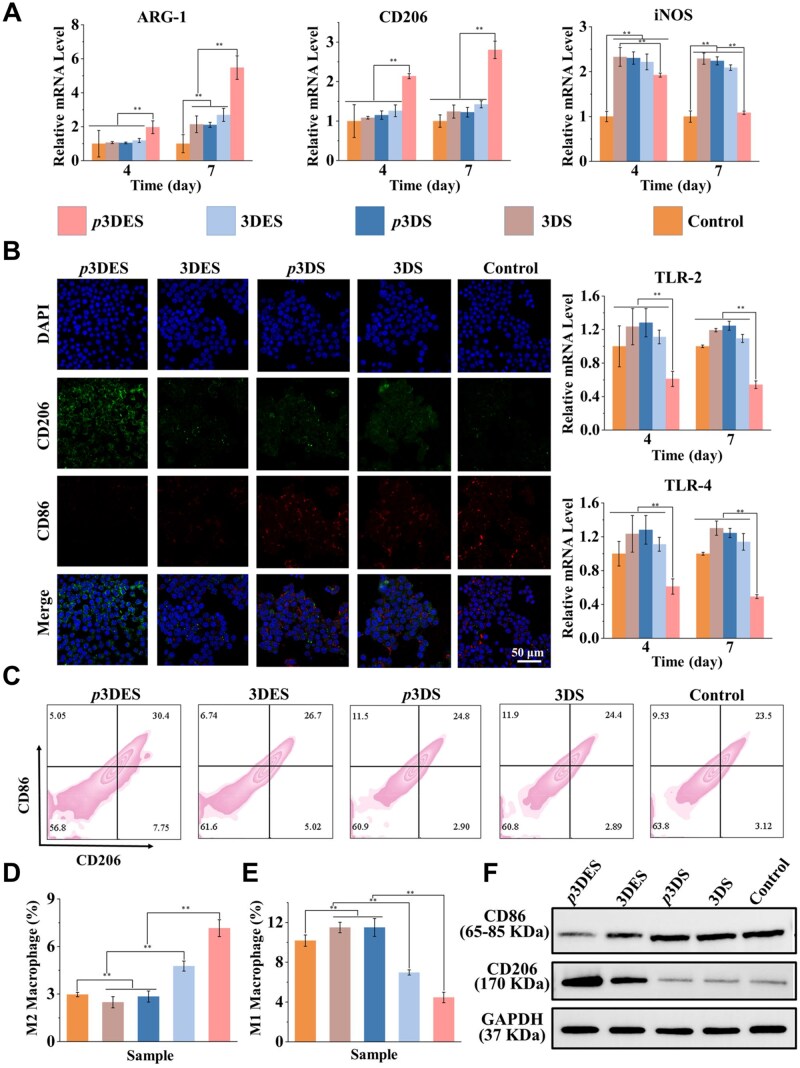
*In vitro* evaluation of macrophage phenotype modulation. (**A**) Expression of phenotype-related genes ARG-1 (M2), CD206 (M2), iNOS (M1), TLR-2 (M1) and TLR-4 (M1) in RAW264.7 cells treated with different scaffolds for 4 and 7 days. (**B**) Immunofluorescence staining results of CD86 (M1) and CD206 (M2) expression in RAW264.7 cells treated with different scaffolds for 2 days. (**C**–**E**) Analysed macrophage polarization by flow cytometry in RAW264.7 cells treated with different scaffolds for 2 days (CD86 represents M1 phenotype macrophages, CD206 represents M2 phenotype macrophages). (**F**) Protein expression of CD86 (M1) and CD206 (M2) in RAW264.7 cells treated with different scaffolds for 4 days. The control group was operated without scaffold treatment. **P < 0.01, highly significant (*n* = 3; error bars represent standard deviation).

Macrophages play a crucial role in regulating the immune microenvironment during the initial stage of bone repair [55]. Research indicates that macrophages can polarize from the M0 or M1 phenotype to the anti-inflammatory M2 phenotype under ES and secrete various cytokines to regulate the initial inflammatory response, stem cell recruitment and subsequent osteogenic differentiation [56]. Therefore, RAW264.7 macrophages were co-cultured with the scaffolds to assess the immunomodulatory effects of the scaffolds. The expression levels of macrophages’ phenotype-related genes are shown in Figure 5A. The results demonstrated that the polarized 3DES exhibited the highest expression level of M2 phenotype-related genes (ARG-1, CD206) and the lowest expression level of M1 phenotype-related genes (iNOS, TLR-2, TLR-4), indicating that the charge carried on the polarized 3DES surface promotes macrophage M2 polarization. In the initial stage of bone repair, macrophages firstly polarize into the M1 phenotype to participate in the initial immune response and then polarize into the M2 phenotype to participate in the bone repair process [57]. Biomaterials with immunomodulatory properties can induce M1 phenotype macrophages to polarize into M2 phenotype, creating an environment conducive to bone repair [58]. According to qPCR results, the polarized 3DES can induce M1 phenotype macrophages to polarize into M2 phenotype, and compared to the other four groups, polarized 3DES exhibits the highest regulatory effect. This result demonstrates that the polarized 3DES has excellent immunomodulatory effects and can provide a beneficial immune microenvironment for bone repair.

Additionally, the macrophage phenotype regulation performance of the scaffolds was further evaluated by immunofluorescence staining, as shown in Figure 5B. Consistent with the qPCR results, the polarized 3DES exhibited the most vigorous green and the weakest red fluorescence, indicating that the polarized 3DES could induce the most significant number of M2 phenotype macrophages. The flow cytometry results in Figure 5C–E indicated that the polarized 3DES could induce the highest number of M2 phenotype macrophages and the lowest number of M1 phenotype macrophages, showing a significant difference compared to the other groups. This result demonstrates the outstanding immunomodulatory capability of the polarized 3DES. Furthermore, western blotting was employed to assess the protein expression levels of CD86 and CD206 in macrophages treated with different scaffolds. Figure 5F shows that the polarized 3DES exhibited the highest CD206 protein expression. Conversely, the density of the CD86 bands in the polarized 3DES was the lowest, suggesting minimal CD86 protein expression. These results confirm that the polarized 3DES possesses the most potent ability to induce M2 polarization of macrophages and can effectively regulate the immune microenvironment.

According to previous studies, immune cells such as macrophages play a crucial role in bone regeneration, especially M2 macrophages [59]. These M2 macrophages can secrete various cytokines that mediate the interaction between immune regulation and osteogenic regeneration [57]. We have confirmed that the polarized 3DES can promote the polarization of macrophages toward the M2 phenotype. Therefore, to further validate the role of macrophages in the bone repair process, the secretion levels of osteogenic markers (BMP-2 and TGF-β1) in macrophages were assessed through immunofluorescence staining. BMP-2 is a crucial growth factor capable of inducing the expression of various osteogenic genes, playing a significant role during the bone repair process. On the other hand, TGF-β1 can promote the proliferation and differentiation of intramembranous osteoblasts [60, 61]. The results are shown in Supplementary Figure S16, where red and green represent BMP-2 and TGF-β1, respectively. The results showed that the cells treated with polarized 3DES exhibited the highest secretion levels of osteogenic markers, confirming that the polarized 3DES can effectively promote macrophage polarization toward the M2 phenotype and secretion of osteogenic cytokines.

Western blotting was used to further analyse the secretion and release of BMP-2 and TGF-β1 in macrophages. RAW264.7 cells were treated with different scaffolds, and the supernatant was collected for western blotting analysis. As shown in Supplementary Figure S17, the polarized 3DES had the highest protein content, which proved that the composite scaffold could stimulate the secretion and release of BMP-2 and TGF-β1 by macrophages through ES.

Subsequently, to evaluate the immunoregulatory role of the composite scaffold in osteogenesis, RAW264.7 cells were treated with different scaffolds, and the resulting conditioned medium was collected to culture BMSCs for ALP and ARS staining (Supplementary Figure S18). The results showed that the polarized 3DES exhibited the highest ALP and ARS staining areas and the darkest color, indicating that the composite scaffold can enhance osteogenesis via immunoregulation.

We have demonstrated that composite scaffolds can induce macrophages to polarize from M0 or M1 phenotype to M2 phenotype and secrete inflammatory and osteogenic factors, regulating the immune microenvironment and promoting stem cell osteogenic differentiation. Previous studies have shown that the activation of the PI3K/Akt signaling pathway can induce polarization of macrophages from the M0 or M1 phenotype to the M2 phenotype [34, 62]. Consequently, the related signaling pathways involved in ES-induced macrophage M2 polarization were investigated through western blotting. The western blotting results in Supplementary Figure S19 showed that the polarized 3DES exhibited the highest protein expression levels of P-PI3K and P-Akt, indicating that the composite scaffolds can effectively induce macrophage M2 polarization by activating the PI3K/Akt signaling pathway.

### *In vitro* osteogenic activity of composite scaffold

**Figure 6.**
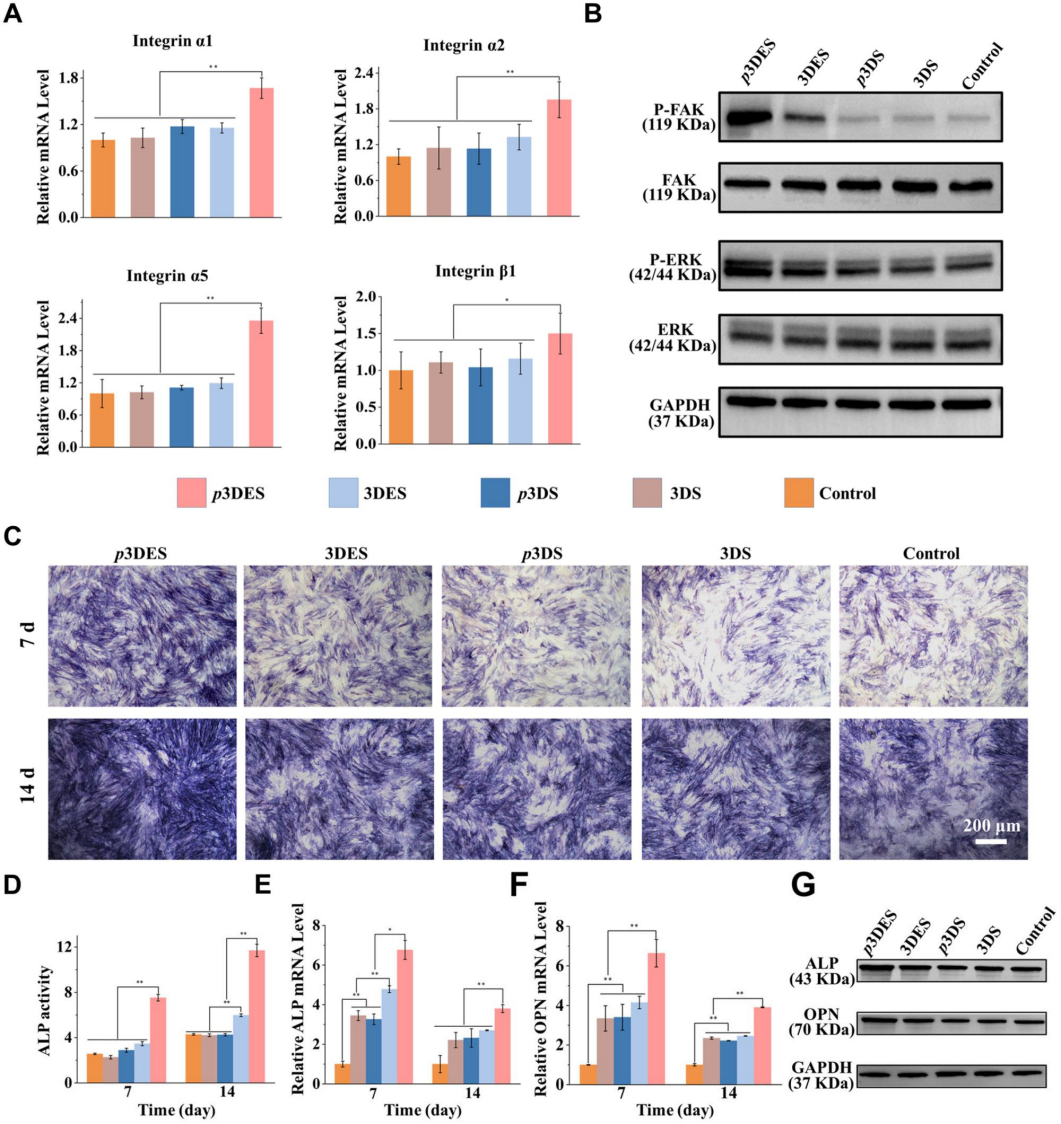
*In vitro* early osteogenic differentiation study. (**A**) Expression of integrin subtypes-related genes in BMSCs treated with different scaffolds after 6 h. (**B**) Expression of osteogenic signaling pathway-related proteins in BMSCs treated with different scaffolds after 24 h. (**C** and **D**) ALP staining results of BMSCs treated with different scaffolds for 7 and 14 days and quantitative analysis of ALP activity. (**E** and **F**) Expression of early osteogenic-related genes in BMSCs treated with different scaffolds for 7 and 14 days. (**G**) Expression of early osteogenic-related proteins in BMSCs treated with different scaffolds for 21 days. The control group was operated without scaffold treatment. *P < 0.05, significant **P < 0.01, highly significant (*n* = 3; error bars represent standard deviation).

Integrin subtypes are a group of cell surface receptors, among which integrin α1, α2, α5 and β1 play a crucial role in stem cell osteogenic differentiation, which can promote the expression of relevant osteogenic genes and proteins by activating the corresponding signaling pathways [63]. The results of integrin subtype-related gene expression are shown in Figure 6A. The BMSCs treated with polarized 3DES exhibited the highest expression levels of integrin α1, α2, α5 and β1, indicating that the polarized 3DES can effectively stimulate the expression of integrin subtypes of BMSCs. Integrin α2β1 is known to lead to the phosphorylation of Runx2 by activating the FAK signaling pathway, while integrin α1β1 can interact with type IV collagen to influence osteogenic differentiation [64, 65]. The integrin α5 can also enhance osteogenic differentiation by activating the FAK/ERK1/2-MAPKs and PI3K signaling pathways [66]. Consequently, the protein expression of P-FAK and its downstream P-ERK was assessed through western blotting. The results shown in Figure 6B indicated that the polarized 3DES exhibited the highest protein expression levels of P-FAK and P-ERK, suggesting that the polarized 3DES can enhance the osteogenic differentiation of BMSCs by activating the FAK/ERK signaling pathway.

Previous studies have demonstrated that ES can directly promote stem cell proliferation and osteogenic differentiation [67]. As an early osteogenic marker, ALP is an essential osteogenic marker for cell maturation and calcification [68, 69]. BMSCs were co-cultured with different scaffolds in an osteogenic induction medium, with ALP staining performed at 7 and 14 days. As shown in Figure 6C, the ALP staining results indicated that the BMSCs treated with polarized 3DES had the largest ALP-stained area, indicating that BMSCs treated with the polarized 3DES exhibited the highest ALP expression levels. The ALP activity assay results (Figure 6D) confirmed that the BMSCs treated with polarized 3DES exhibited the highest ALP activity, consistent with the ALP staining results. The early osteogenic-related gene expression of ALP and OPN was determined by qPCR. RNA was extracted for qPCR analysis after BMSCs were treated with different scaffolds for 7 and 14 days. Figure 6E and F shows that BMSCs treated with polarized 3DES exhibited the highest early osteogenic markers (ALP and OPN) expression levels. Subsequently, early osteogenesis-related protein expression was assessed using western blotting. As shown in Figure 6G, for the two target proteins ALP and OPN, BMSCs treated with polarized 3DES exhibited the highest protein expression levels. These results indicate that the polarized composite scaffolds can promote early osteogenic differentiation of stem cells by activating osteogenesis-related signaling pathways.

### *In vitro* extracellular matrix mineralization of composite scaffold

**Figure 7.**
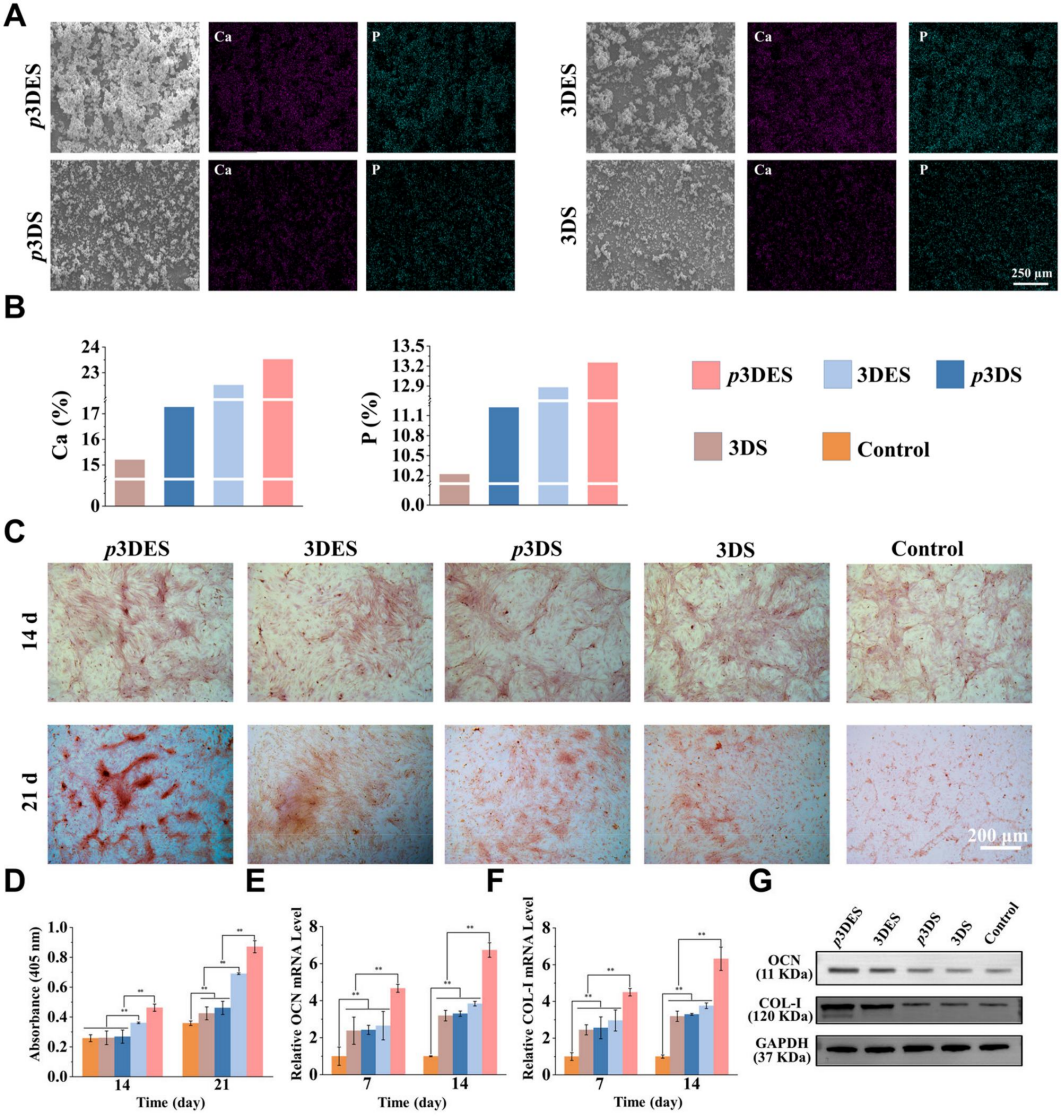
*In vitro* late extracellular matrix mineralization study. (**A**) SEM image and elemental mapping of surface mineralization components of polarized and non-polarized scaffolds after simulated body fluid treatment for 7 days. (**B**) The content of calcium and phosphorus ions in the surface mineralization components of polarized and non-polarized scaffolds after simulated body fluid treatment for 7 days. (**C**) ARS staining results of BMSCs treated with different scaffolds for 14 and 21 days. (**D**) Quantitative analysis of ARS activity. (**E** and **F**) Expression of late osteogenic-related genes in BMSCs treated with different scaffolds for 7 and 14 days. (**G**) Expression of late osteogenic-related proteins in BMSCs treated with different scaffolds for 21 days. The control group was operated without scaffold treatment. **P < 0.01, highly significant (*n* = 3, error bars represent standard deviation).

The increased concentration of ions such as calcium and phosphate in the physiological environment can effectively promote bone formation [70, 71]. Under physiological conditions, biomaterials with the capacity for surface mineralization can form a mineralized matrix on their surfaces, which can continuously supply calcium and phosphate ions to promote osteogenic differentiation of stem cells and extracellular matrix mineralization [72]. In the late stage of bone defect repair, cations such as calcium are crucial for the mineralization of extracellular matrix [33]. After osteogenic differentiation, stem cells will secrete bone matrix proteins to form an extracellular matrix, then the calcium ions near the extracellular matrix combine with phosphate ions to form inorganic salts and deposit into the extracellular matrix, ultimately promoting the mineralization of the extracellular matrix [32, 33]. According to the piezoelectric characterization results, we know that the surface of the composite scaffold carries a negative charge after polarization. Under physiological conditions, the composite scaffold can attract cations such as calcium to generate mineralized substances, which are essential for the final bone defect repair process [73]. In this study, both polarized and non-polarized scaffolds were immersed in a 1.5-fold simulated body fluid (1.5 × SBF). After 7 days, the scaffolds were retrieved, cleaned and dried, and the surface mineralization components were observed and analysed using SEM and elemental mapping. As shown in Figure 7A, SEM results revealed that the polarized 3DES exhibited the thickest mineralization layer. In contrast, the mineralization layer thickness of the non-polarized 3DES was slightly higher than that of the polarized 3DS and non-polarized 3DS. This trend is consistent with the piezoelectric performance characterization results, indicating that the addition of CMBT short nanofibers enhances the inducible mineralization capability of 3DS, and the polarized 3DES has the most robust inducible mineralization ability. The elemental analysis results showed that the polarized 3DES had the highest Ca and P element contents (Figure 7B and Supplementary Figure S20). The Ca/P ratio of the mineralized layer in the polarized 3DES was closest to that of hydroxyapatite (approximately 1.7), which further confirmed that the polarized 3DES had the most surface charges and was able to attract the most ions to the scaffold surface to form a mineralized matrix [74]. The XRD results (Supplementary Figure S21) indicated that the polarized 3DES’s mineralized layer exhibited a typical characteristic peak of hydroxyapatite, further confirming the superior inducible mineralization capability of 3DES. These results demonstrated that the polarized composite scaffolds can adsorb calcium and phosphate ions to form a mineralized matrix, providing necessary ions such as calcium and phosphate for extracellular matrix mineralization during late osteogenesis.

Next, the extracellular matrix mineralization performance of the composite scaffolds at the late stage of osteogenesis was assessed. BMSCs were treated with different scaffolds and underwent ARS staining at 14 and 21 days. As shown in Figure 7C, the results showed that BMSCs treated with the polarized 3DES exhibited the largest ARS-stained area. This result indicates that the polarized 3DES can induce the formation of a more significant amount of mineralized extracellular matrix, demonstrating the excellent osteogenic differentiation effect. Quantitative analysis results of ARS (Figure 7D) also indicated that the polarized 3DES exhibited an excellent extracellular matrix mineralization effect.

Late osteogenic-related gene (OCN and COL-I) expression was assessed using qPCR. RNA was extracted for qPCR analysis after BMSCs were treated with different scaffolds for 7 and 14 days. As shown in Figure 7E and F, the results showed that BMSCs treated with polarized 3DES exhibited the highest expression levels of the late osteogenic-related genes (OCN and COL-I). These results indicate that the polarized composite scaffolds can effectively promote the expression of late osteogenic-related genes. Subsequently, late osteogenic-related protein expression was assessed using western blotting. As shown in Figure 7G, for the two target proteins OCN and COL-I, BMSCs treated with polarized 3DES exhibited the highest protein expression levels, indicating that the polarized composite scaffolds have the strongest osteogenic properties. After electric field polarization, the surface of 3DES carries a large amount of charge, promoting the expression of osteogenic genes and proteins. Additionally, the surface charge on the scaffolds can enhance the adsorption capacity for water molecules and ions, thereby promoting extracellular matrix mineralization [73]. In summary, the charge generated by the polarized scaffolds can facilitate both the early osteogenic differentiation and late extracellular matrix mineralization of BMSCs, making it a promising platform for bone regeneration.

### *In vivo* evaluation of rat cranial bone defect repair

**Figure 8.**
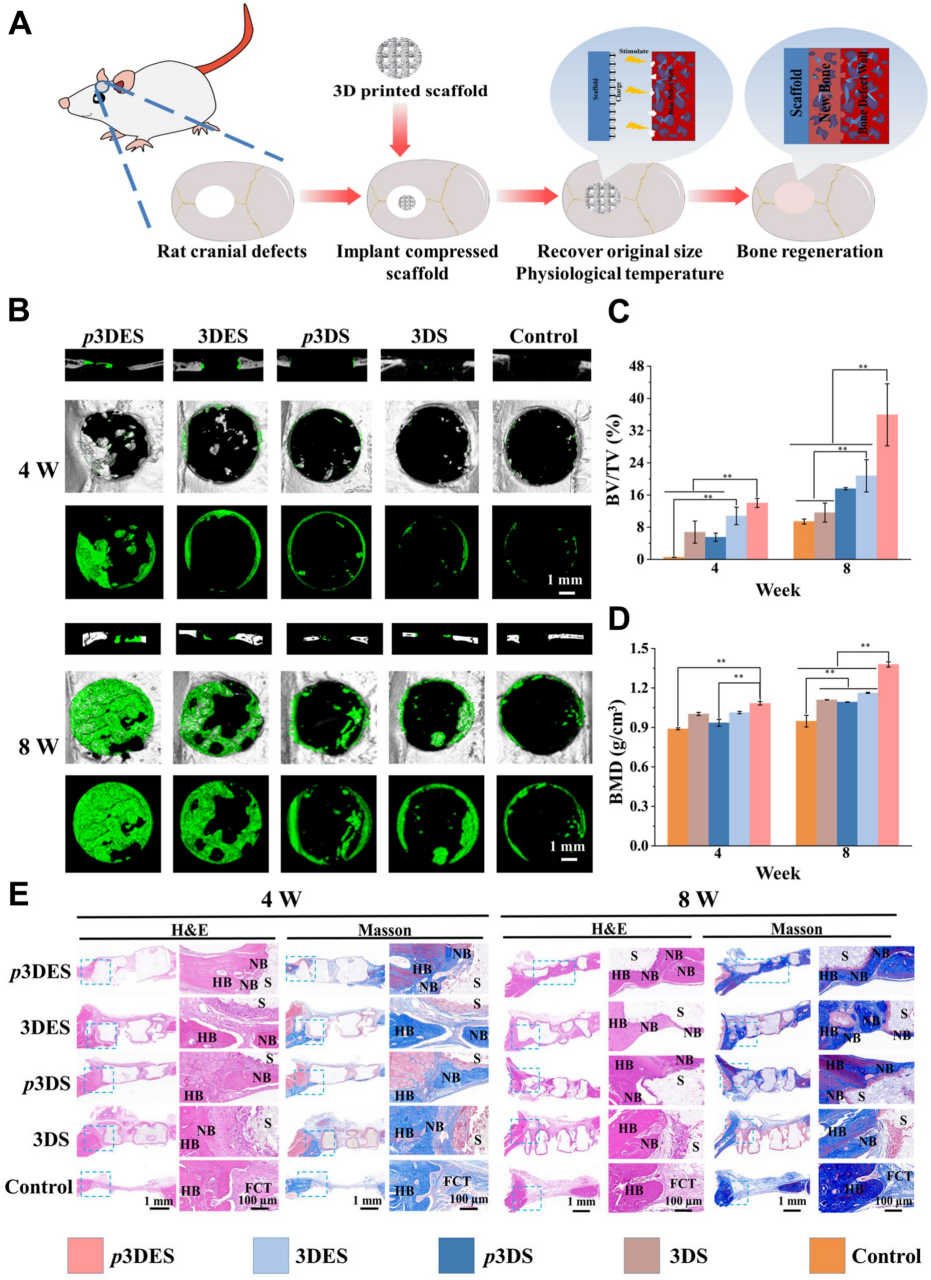
*In vivo* evaluation of bone defect repair with scaffolds. (**A**) Schematic diagram of the scaffold implantation procedure. (**B**–**D**) Reconstructed 3D micro-CT images of the rat skull and the regenerated bone’s BV/TV and BMD values. (**E**) Histological evaluation using H&E staining and Masson trichrome staining. S indicates the location of the residual scaffold, FCT indicates fibrous connective tissue, HB indicates host bone and NB indicates new bone. The control group was operated without scaffold treatment. **P < 0.01, highly significant (*n* = 3, error bars represent standard deviation).

We have demonstrated that the polarized composite scaffold can simultaneously participate in immune regulation, stem cell osteogenic differentiation and extracellular matrix mineralization through *in vitro* cell experiments, preliminarily demonstrating its multistage osteogenic activity. Next, the composite scaffold’s *in vivo* bone defect repair efficacy was evaluated through a rat cranial bone defect repair experiment. The scaffold implantation process is illustrated in Figure 8A; a round cranial bone defect with a diameter of 5 mm was created, and subsequently, the compressed scaffold was implanted in the defect site. Due to its ability to be compressed into a smaller size, the scaffold could be easily implanted into the defect site. After implantation, the scaffold could rapidly recover to its original size and fill the defect site.

The generation of new bone was assessed using micro-CT scans. Figure 8B shows the reconstructed micro-CT sagittal and top-down views, respectively. The results indicated that even at 8 weeks post-implantation, the control group exhibited minimal new bone generation. Non-polarized 3DES and 3DS, as well as polarized 3DS, showed only a small amount of new bone generation at the periphery, and due to insufficient osteogenic capability, the thickness of the generated new bone was low. In contrast, the polarized 3DES revealed significant new bone generation, with the newly generated bone almost completely covering the defect site. This result suggests that the polarized 3DES exhibits a more robust bone repair effect.

The bone volume fraction (BV/TV) and bone mineral density (BMD) values obtained through micro-CT analysis are presented in Figure 8C and D. At the 8-week post-implantation, the polarized 3DES exhibited the highest BV/TV and BMD values, indicating that the surface charge carried by the 3DES after polarization significantly promotes bone regeneration at the defect site.

The histological staining evaluation was conducted on decalcified sections of the cranial bones. The H&E and Masson’s trichrome staining results are illustrated in Figure 8E. The H&E staining result revealed no signs of inflammation, fibrotic reactions or pathological abnormalities at the defect sites treated with various scaffolds. Notably, the polarized 3DES displayed the highest level of new bone formation. In contrast, polarized 3DS, non-polarized 3DS, 3DES and the control group showed less new bone formation. Masson’s trichrome staining result indicated that the polarized 3DES had the highest collagen deposition and new bone formation. In contrast, polarized 3DS, non-polarized 3DS, 3DES and the control group displayed only a small amount of new bone formation at the defect edges. After implantation of polarized 3DES into the defect site, the surface charges can recruit BMSCs and stimulate BMSCs to undergo osteogenic differentiation [75]. H&E-stained images of various organs, including the heart, liver, spleen, lungs and kidneys, are illustrated in Supplementary Figure S22. The results confirmed the absence of inflammatory reactions in all examined organs, suggesting that the scaffold possesses excellent *in vivo* biocompatibility.

*In vitro* osteogenic experiments demonstrate that the polarized 3DES can activate the FAK/ERK signaling pathway, inducing BMSCs to secrete osteogenic-related genes and proteins. Therefore, immunofluorescence and immunohistochemistry staining experiments were employed to assess the *in vivo* expression levels of osteogenic markers (OPN and OCN). The immunofluorescence staining results are shown in Supplementary Figure S23A–F, where green represents OCN and red represents OPN. The results indicated that polarized 3DES exhibited the highest fluorescence intensity, indicating the highest expression level of OPN and OCN. The immunohistochemistry results are shown in Supplementary Figure S23G, where the brown regions indicate positive protein expression. The results showed that the polarized 3DES exhibited the highest brown staining areas. In contrast, the non-polarized 3DES, 3DS, polarized 3DS and the control group exhibited limited brown staining areas. This result suggests that the polarized 3DES can effectively stimulate the osteogenic marker expression, indicating excellent osteogenic activity. The above results suggest that the composite scaffold exhibits excellent *in vivo* biocompatibility and bone regeneration capabilities.

Research indicates that the early-stage inflammatory response following scaffold implantation is crucial for bone repair [76]. Prolonged inflammation can significantly retard the bone healing rate, ultimately leading to suboptimal repair outcomes [77]. During the initial stages of inflammation, the transformation of macrophage phenotypes plays a vital role in mitigating subsequent inflammation and promoting bone repair [56]. So, we assessed the macrophage phenotype transformation through immunofluorescence and immunohistochemistry staining, and the expression of iNOS and CD206 were used to identify the M1 and M2 phenotypes of macrophage. The immunofluorescence staining results are shown in Supplementary Figure S24A–F, and the red and green represent iNOS and CD206, respectively. The results showed that the polarized 3DES exhibited the most intense green fluorescence intensity and the weakest red fluorescence intensity, indicating the highest expression level of CD206 and the lowest expression level of iNOS. This result suggests that the polarized 3DES could induce a more significant amount of M2 phenotype macrophage. The immunohistochemistry results are shown in Supplementary Figure S24G, where the brown regions indicate positive protein expression of iNOS and CD206. The results showed that the polarized 3DES had the highest expression level of CD206 and the lowest expression level of CD86, suggesting that the polarized 3DES did not induce significant inflammation and exhibited good biocompatibility. After polarization by an electric field, the scaffold’s surface carries a considerable amount of charge, which can stimulate the transformation of macrophages into the M2 phenotype, thereby alleviating the inflammation during the initial stages of scaffold implantation, facilitating the subsequent recruitment and osteogenic differentiation of stem cells [52, 67]. The above results indicate that the polarized 3DES can promote the transformation of macrophages into the M2 phenotype, alleviating the inflammatory response during the initial stages of scaffold implantation, which is favorable for the subsequent bone repair process. In summary, we have demonstrated that the scaffolds possess multistage osteogenic activity and can simultaneously participate in early immune microenvironment regulation, stem cell osteogenic differentiation and late extracellular matrix mineralization through ES, exhibiting excellent bone regeneration performance.

## Conclusion

In this study, a simple and universal strategy based on 3D printing technology was used to develop electroactive scaffolds with multistage osteogenic activity. The prepared scaffold possesses excellent shape memory properties and is expected for minimally invasive implantation, which is beneficial for clinical application. After electric field polarization, the scaffold’s surface carries a large amount of charge, enabling the scaffold to actively participate in early immune microenvironment regulation, stem cells osteogenic differentiation and late-stage extracellular matrix mineralization, achieving multistage osteogenic activity. Overall, this work provides a novel strategy for constructing bone defect repair scaffolds with multistage osteogenic activity. The development of this innovative strategy will accelerate the innovation of future bone repair materials, further promoting the clinical application of bone tissue engineering.

## Supplementary Material

rbaf010_Supplementary_Data

## Data Availability

The datasets generated and/or analysed during the current study are not publicly available but are available from the corresponding author at a reasonable request.
