# Supplementary material for: 3D printed scaffolds with multistage osteogenic activity for bone defect repair
Source: Regen Biomater. 2025 Mar 10;12:rbaf010. doi: 10.1093/rb/rbaf010 (PMC11947418; doi:10.1093/rb/rbaf010)
Supplement: rbaf010_Supplementary_Data [file rbaf010_supplementary_data.docx]

**3D Printed Scaffolds with** **Multistage Osteogenic Activity for Bone Defect Repair**

Bing Li^1^, Yichao Ma^2^, Kanwal Fatima^1^, Xiaojun Zhou^1^, Shuo Chen^1, 3 *^, Chuanglong He^1 *^

^1^State Key Laboratory for Modification of Chemical Fibers and Polymer Materials, College of Biological Science and Medical Engineering, Donghua University, Shanghai 201620, P. R. China.
^2^Department of Orthopedics, Shanghai General Hospital, Shanghai Jiao Tong University School of Medicine, Shanghai, 200080, China

^3^State Key Laboratory of Molecular Engineering of Polymers, Fudan University, Shanghai, 200433, China.

^*^Correspondence address. E-mail: [schen@dhu.edu.cn](mailto:schen@dhu.edu.cn) (Shuo Chen); [hcl@dhu.edu.cn](mailto:hcl@dhu.edu.cn) (Chuanglong He)


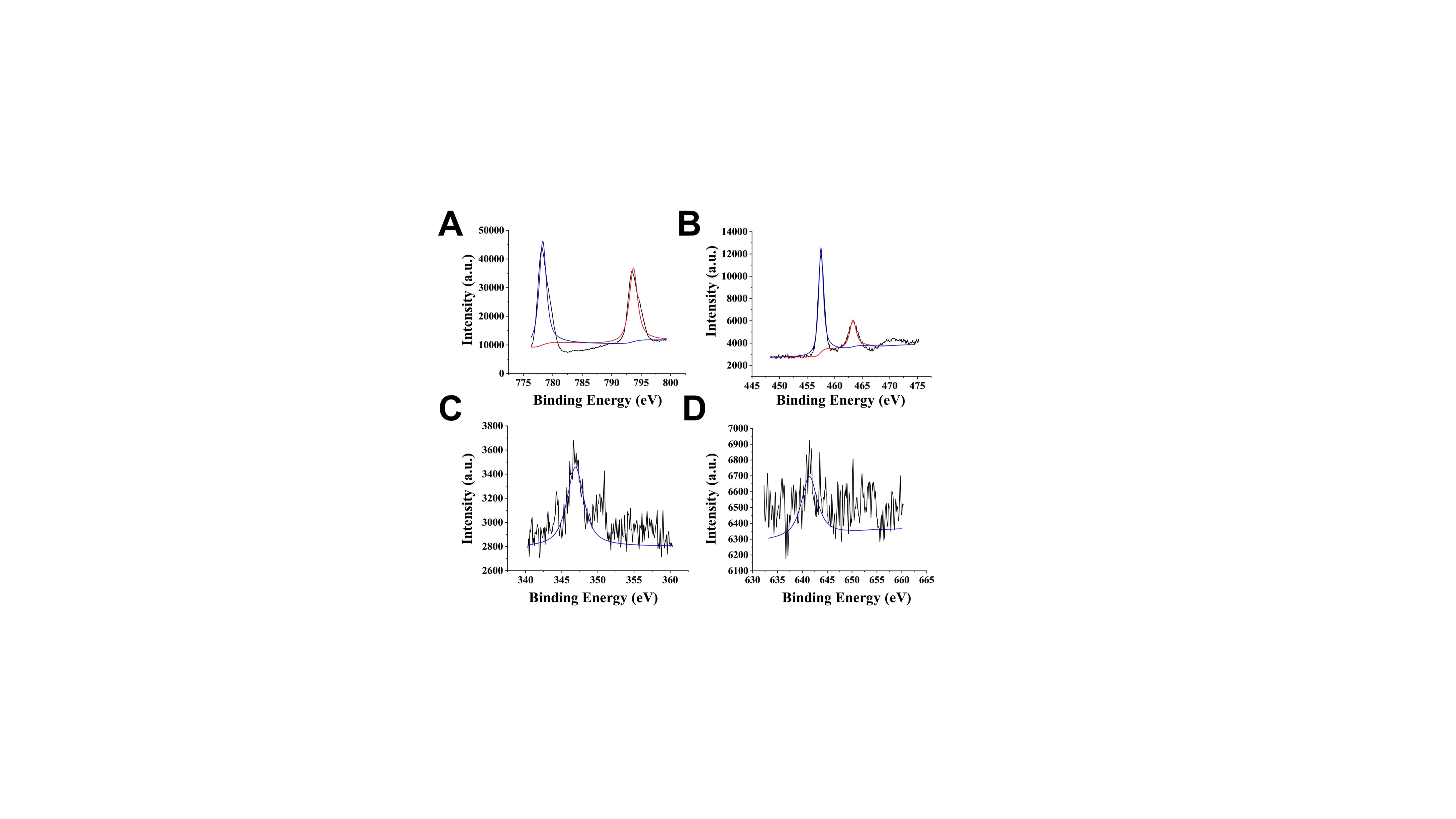


**Supplementary Figure 1**. XPS peak-differentiating and fitting curves: (A) Ba 3d; (B) Ti 2p; (C) Ca 2p; (D) Mn 2p.


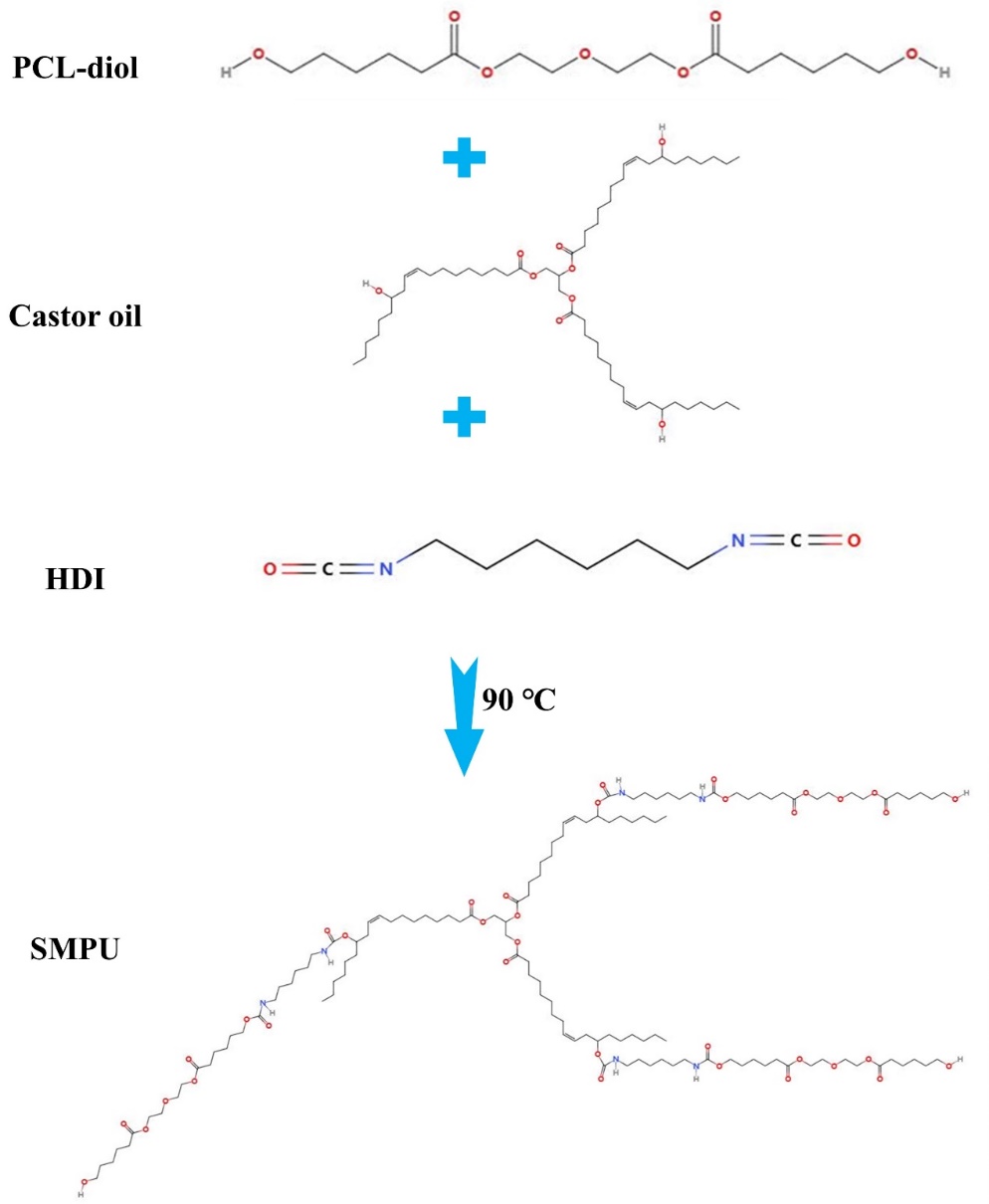


**Supplementary Figure 2**. The preparation process of SMPU.


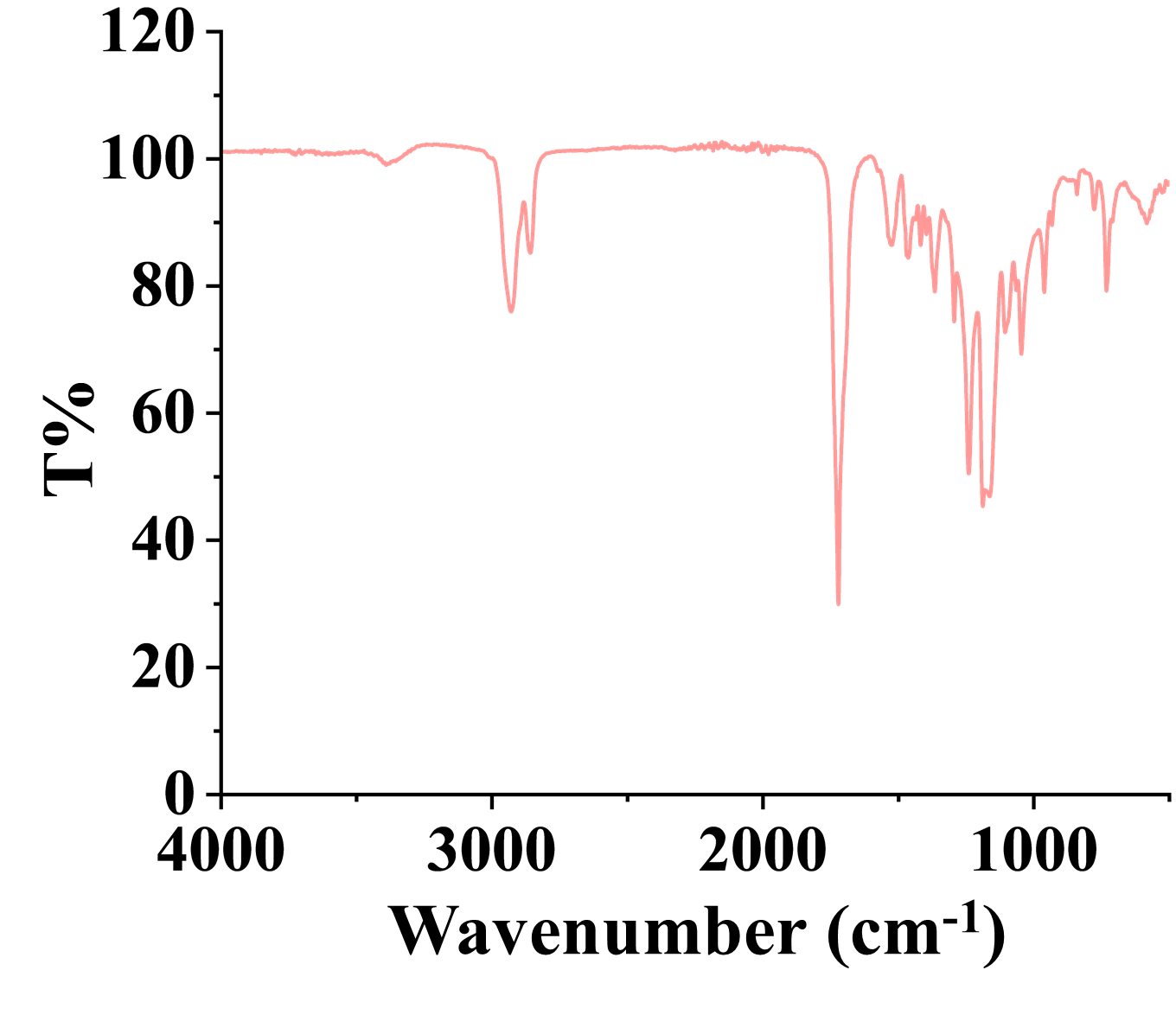


**Supplementary Figure 3**. The FT-IR spectrum of SMPU.


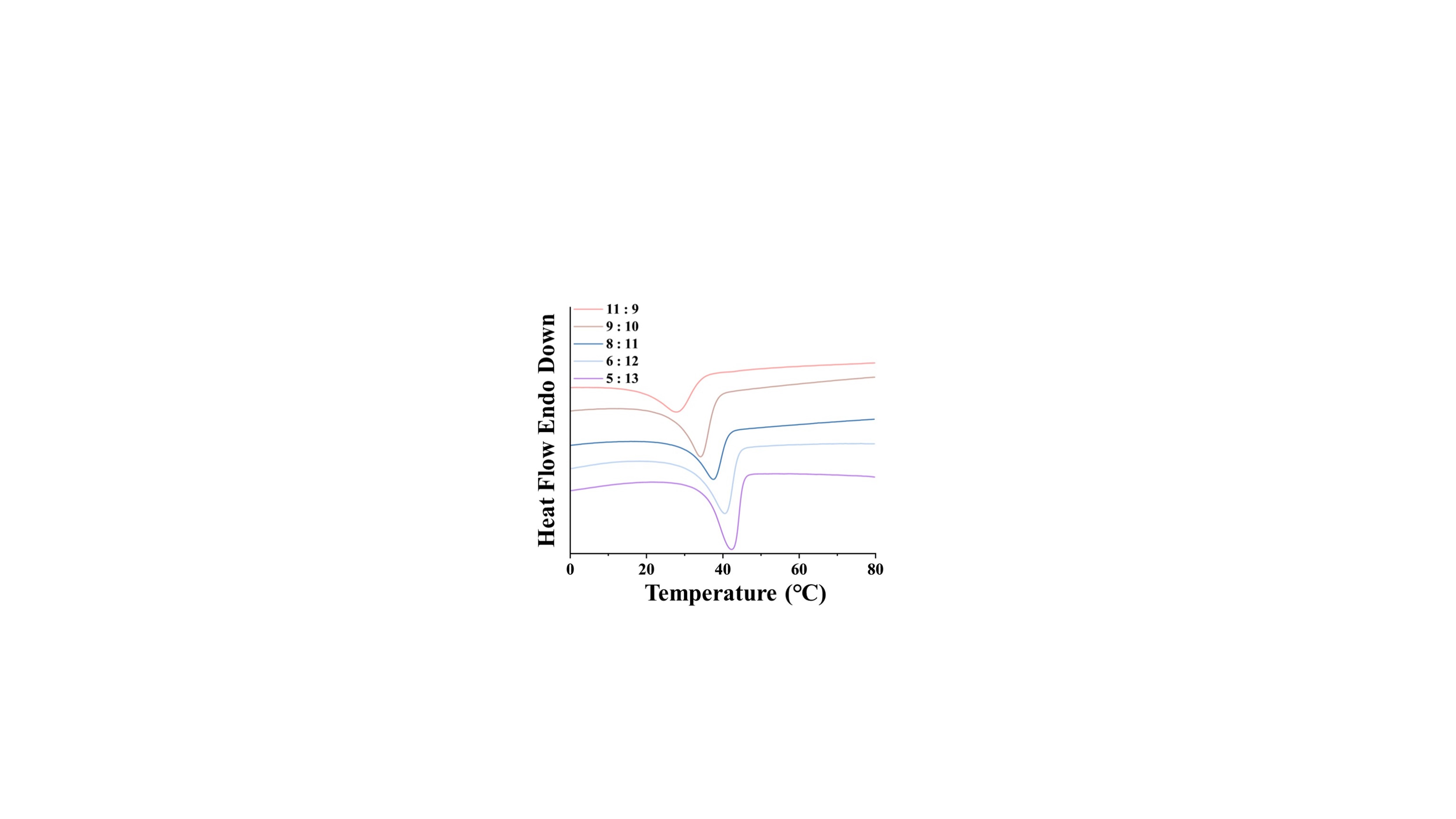


**Supplementary Figure 4**. The DSC curves of SMPU with different PCL: castor oil molar ratio.


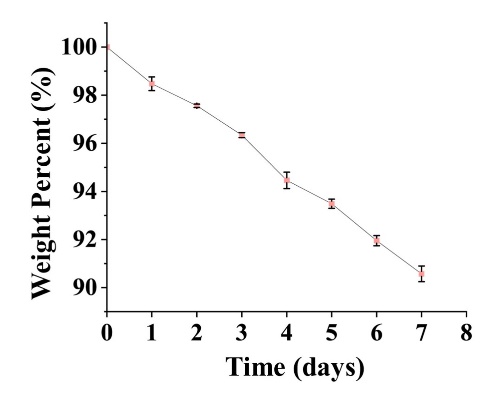


**Supplementary Figure 5**. The degradation behavior of the SMPU over 7 days. (n = 3; error bars represent standard deviation).


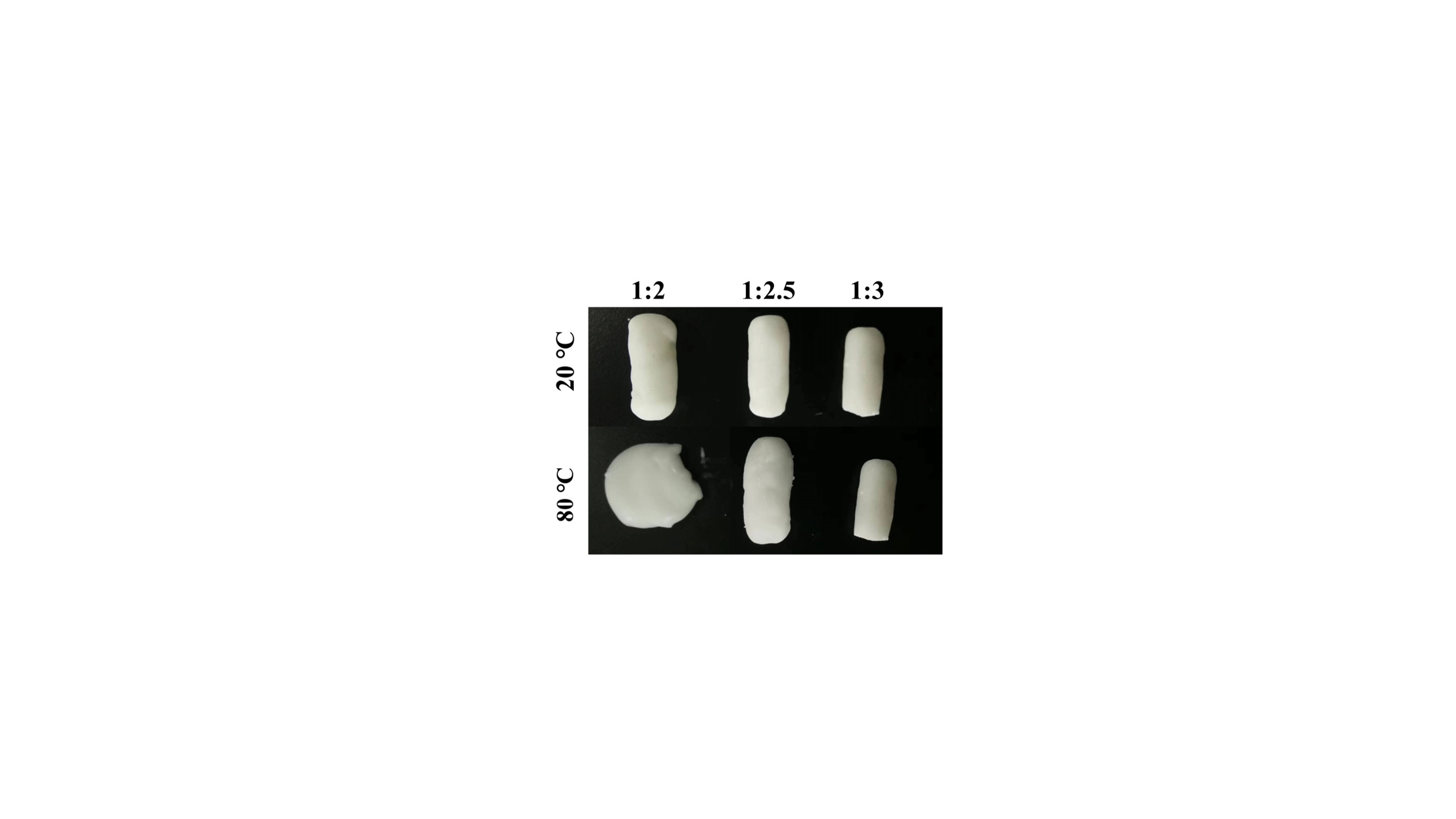


**Supplementary Figure 6**. Exploring ink ratios for scaffold printing.


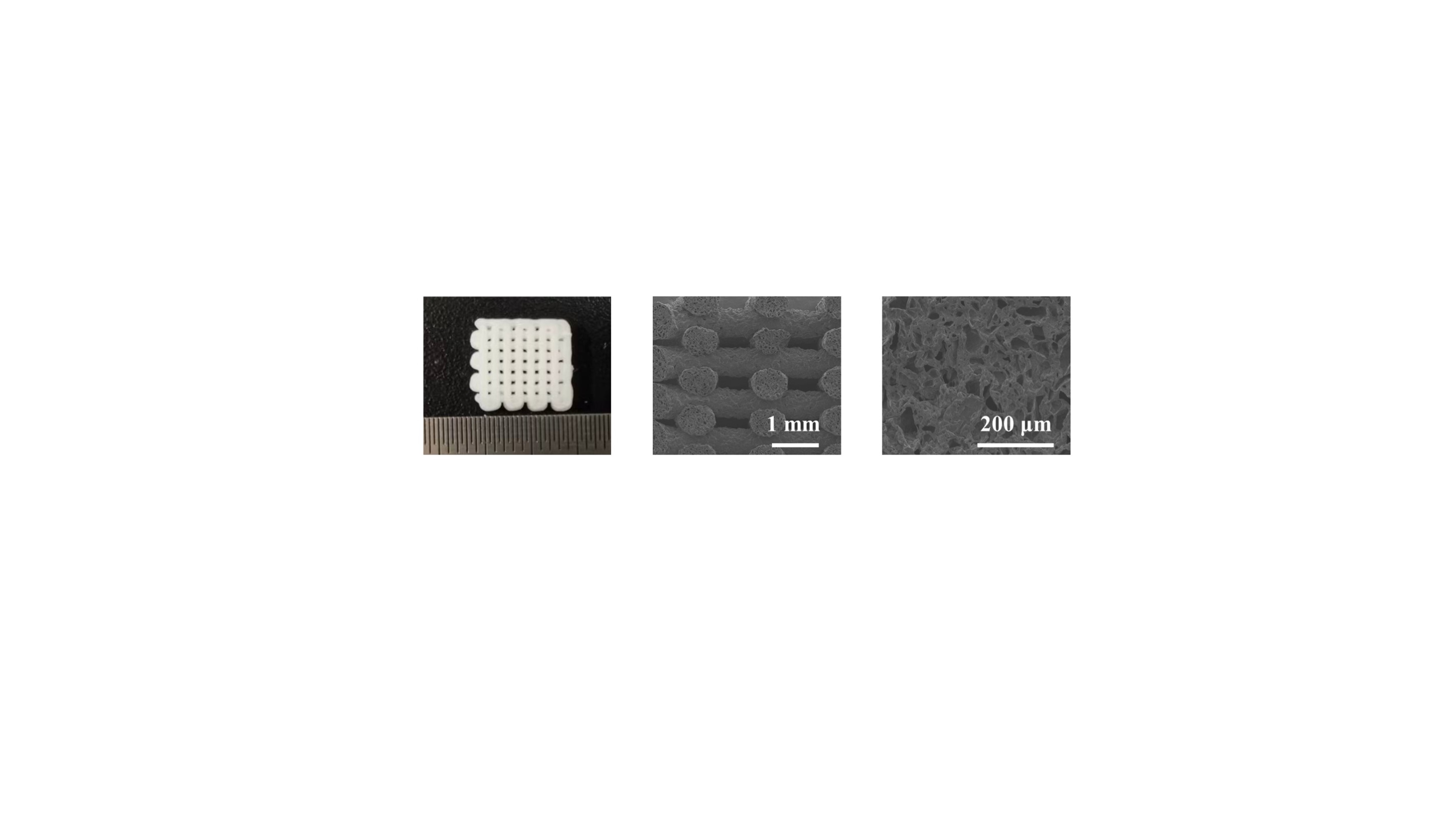


**Supplementary Figure 7**. Macroscopic and microscopic images of 3DS after removal of sodium chloride. (n = 3; error bars represent standard deviation).


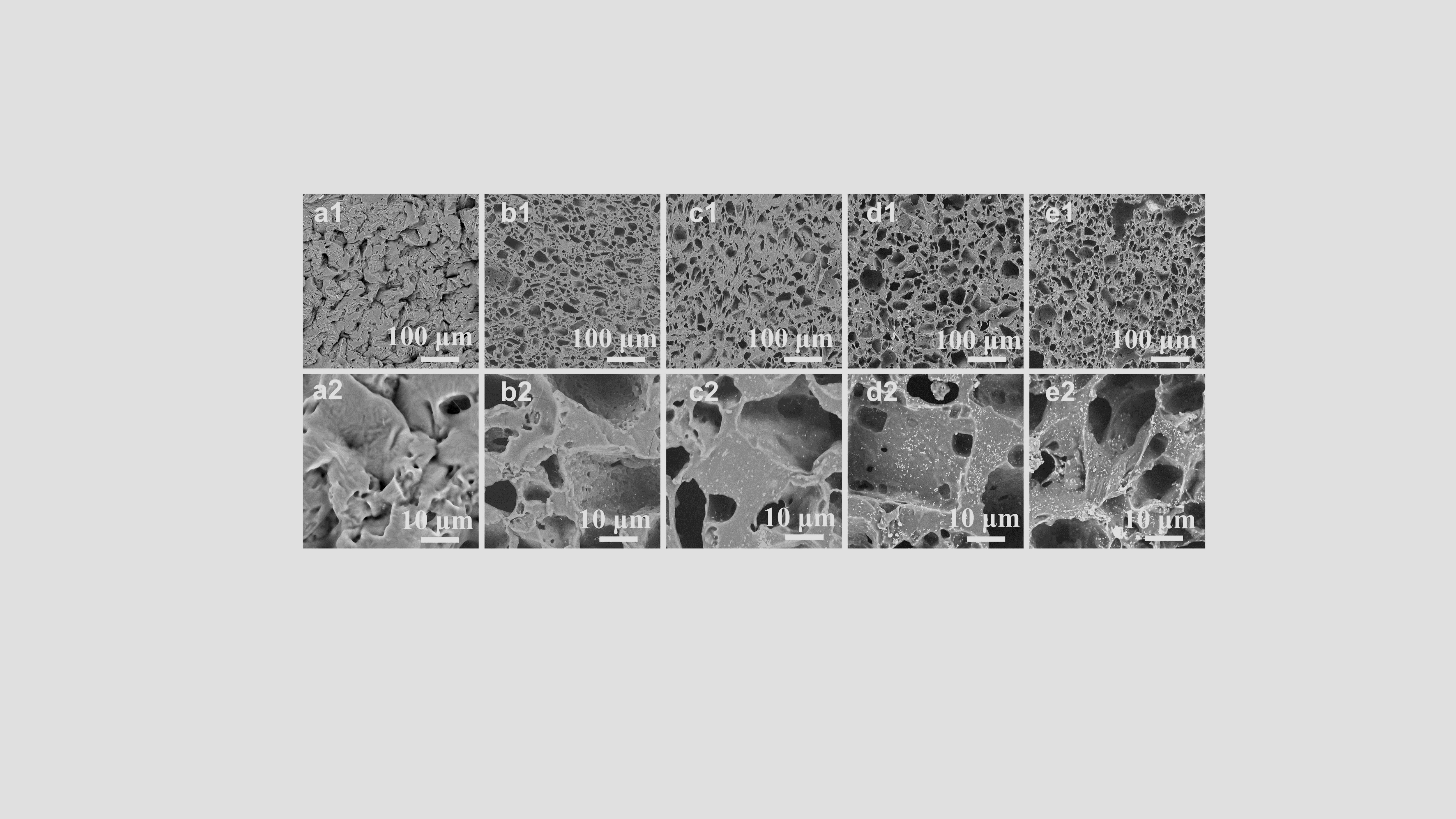


**Supplementary Figure 8**. SEM images of composite scaffolds with different CMBT short nanofiber contents (a1-a2: 0 wt%;b1-b2: 5 wt%; c1-c2: 10 wt%; d1-d2: 20 wt%; e1-e2: 30 wt%).


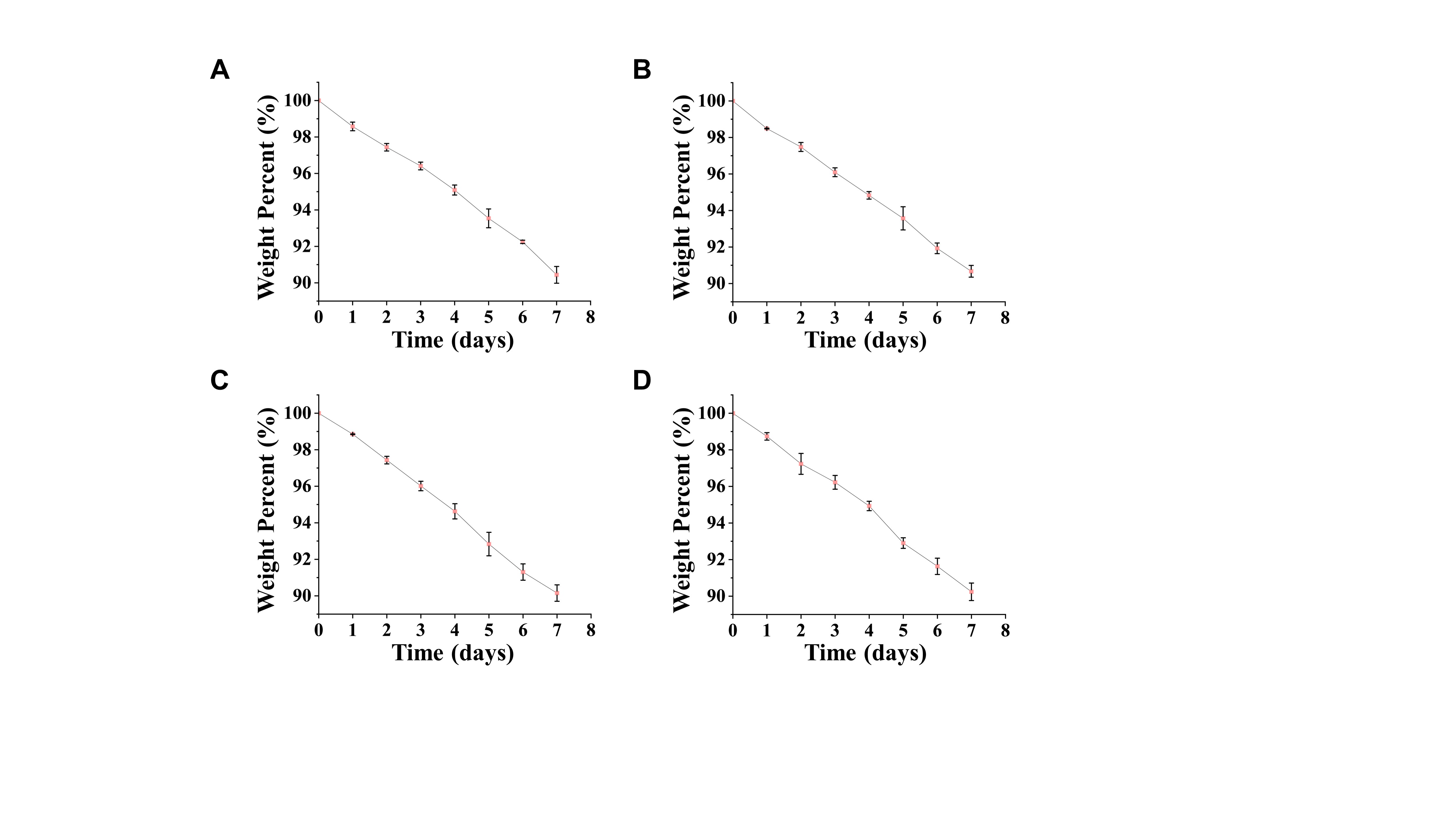


**Supplementary Figure 9**. The degradation behavior of composite scaffolds with different CMBT short nanofiber contents (A-D: 5 wt%, 10 wt%, 20 wt%, and 30 wt%). (n = 3; error bars represent standard deviation).


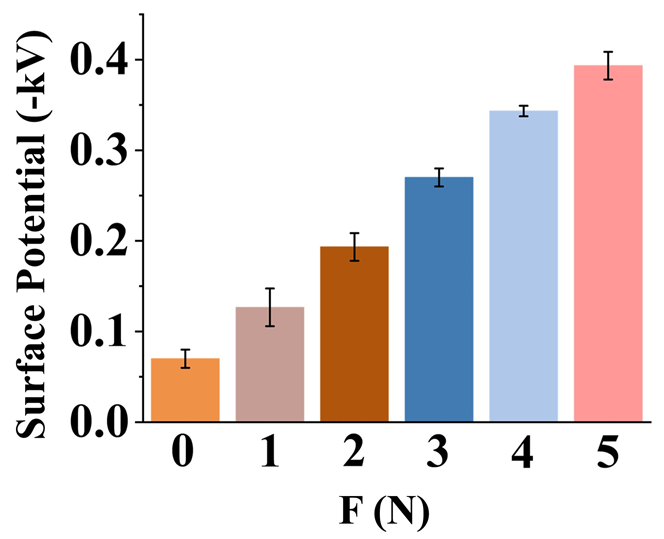


**Supplementary Figure 10**. The surface static voltage of composite scaffold (30 wt%) under different pressures. (n = 3; error bars represent standard deviation).


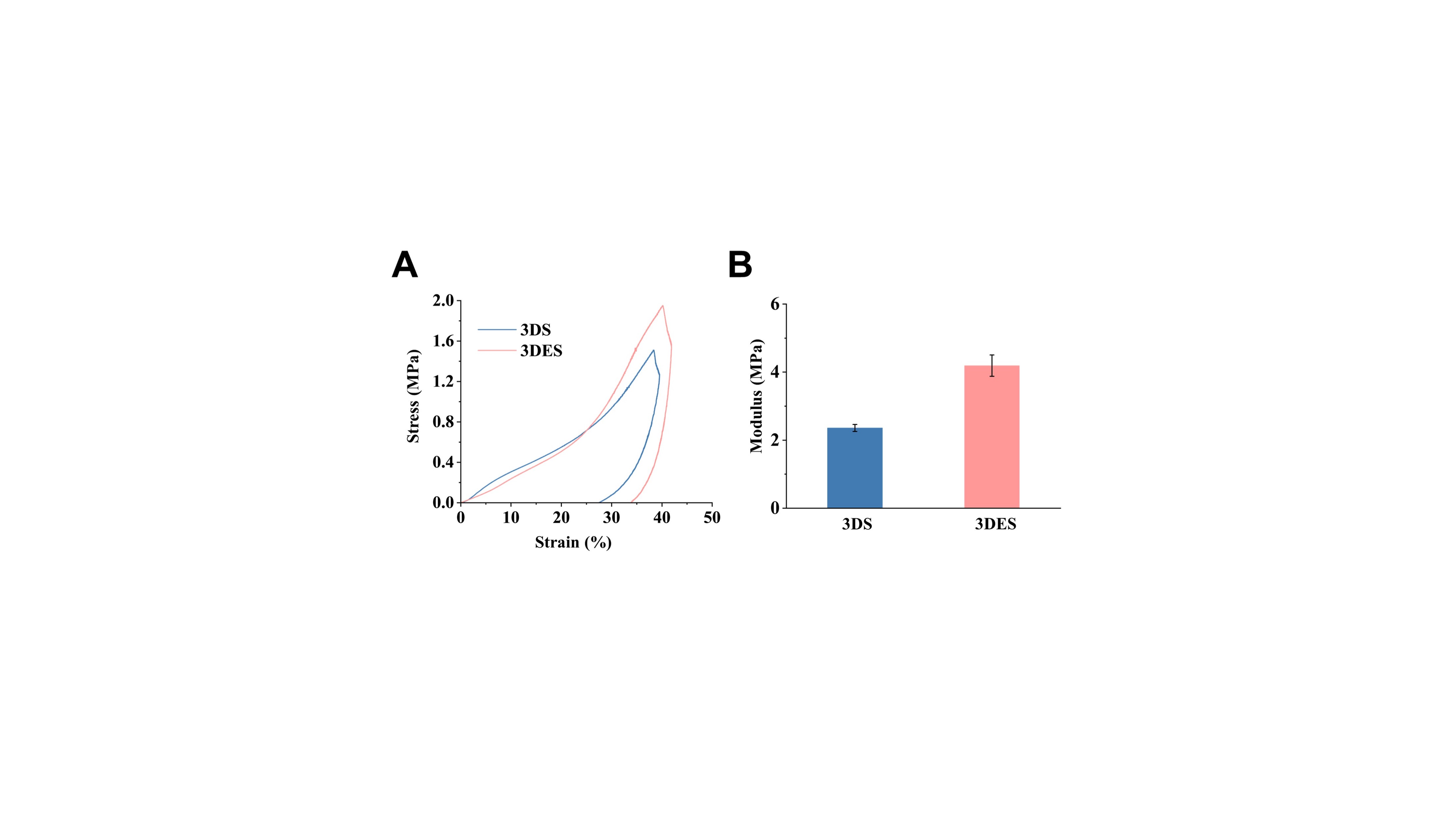


**Supplementary Figure 11**. The mechanical properties of the scaffolds. (A): Stress-strain curve of the scaffolds; (B): The compression modulus of the scaffolds. (n = 3; error bars represent standard deviation).


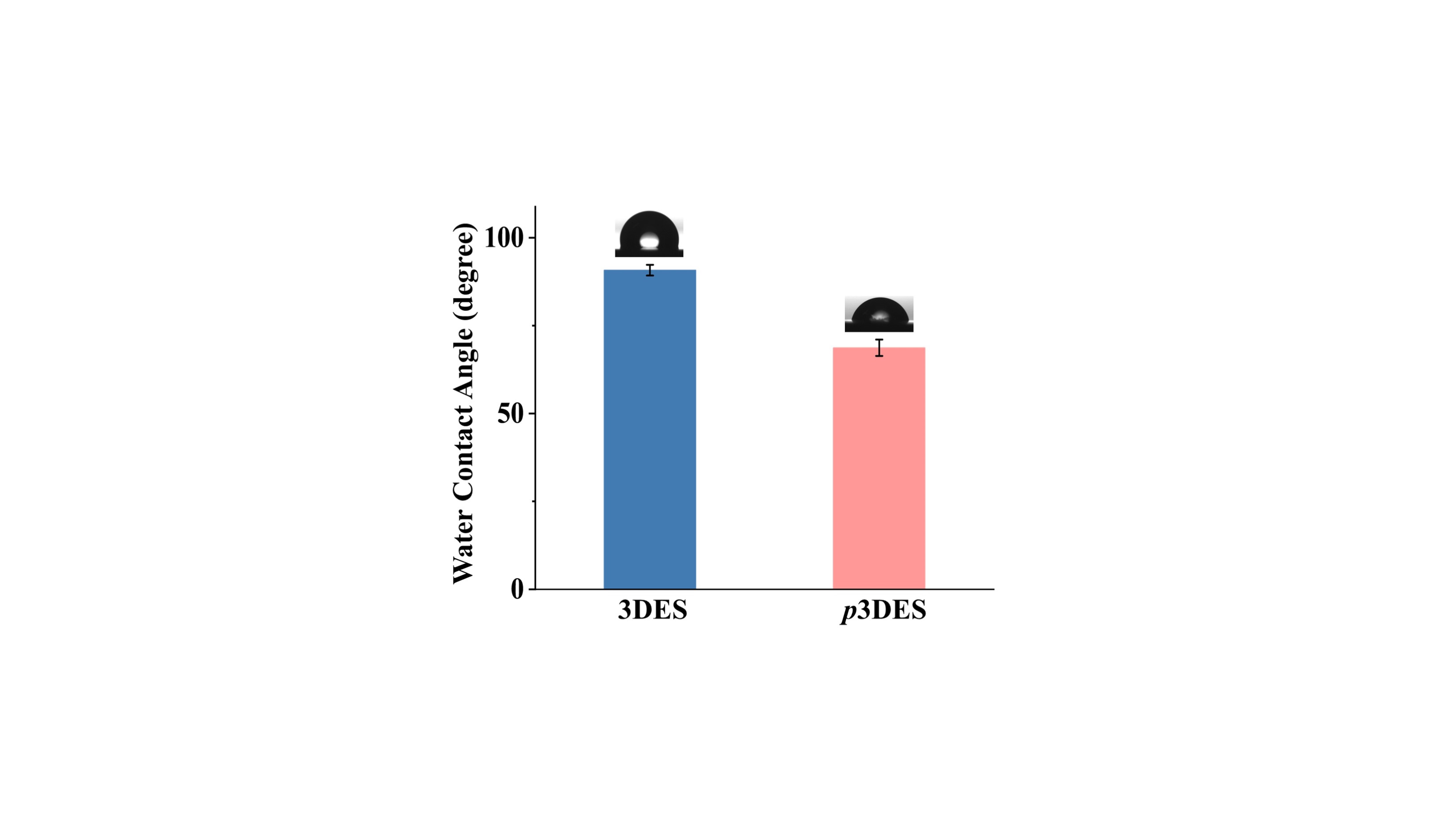


**Supplementary Figure 12**. The water contact angle results of the scaffolds. (n = 3; error bars represent standard deviation).


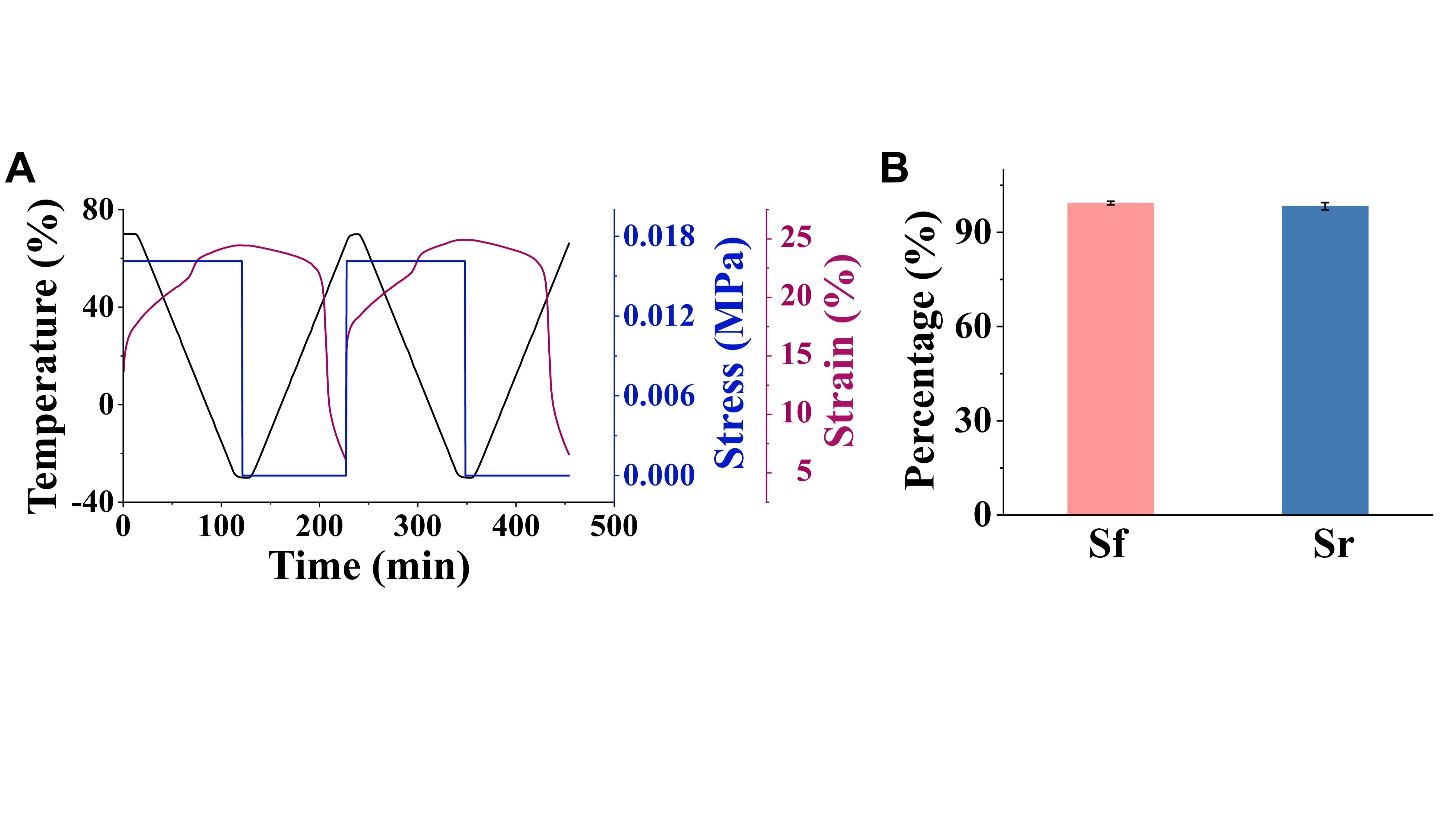


**Supplementary Figure 13**. (A) The shape memory cycle of 3DS; (B) The shape fixed rate (Sf) and shape recovery rate (Sr) of 3DS. (n = 3; error bars represent standard deviation).


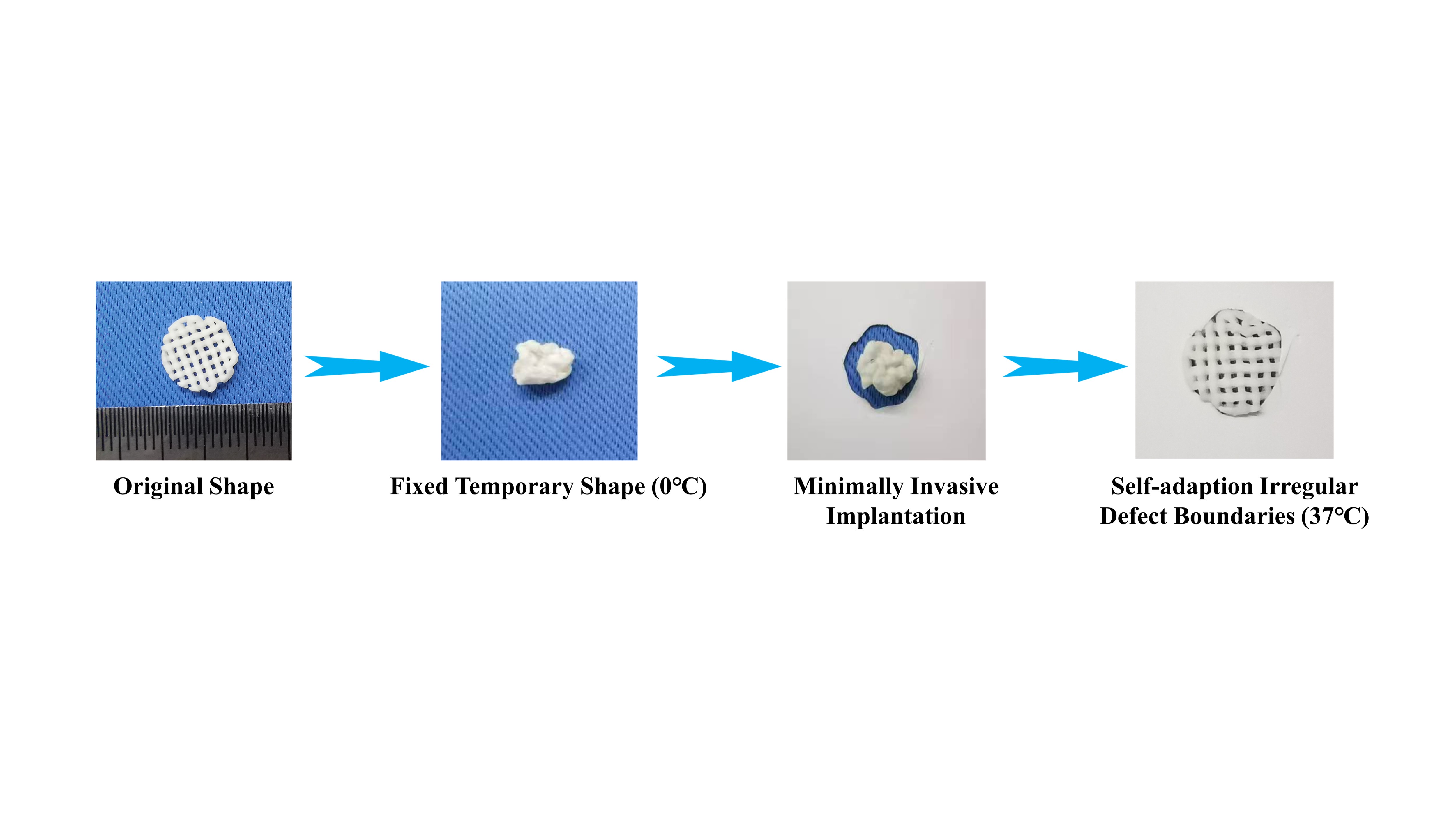


**Supplementary Figure 14**. In vitro simulation of minimally invasive implantation and adaptive bone defect boundary process of 3DES.


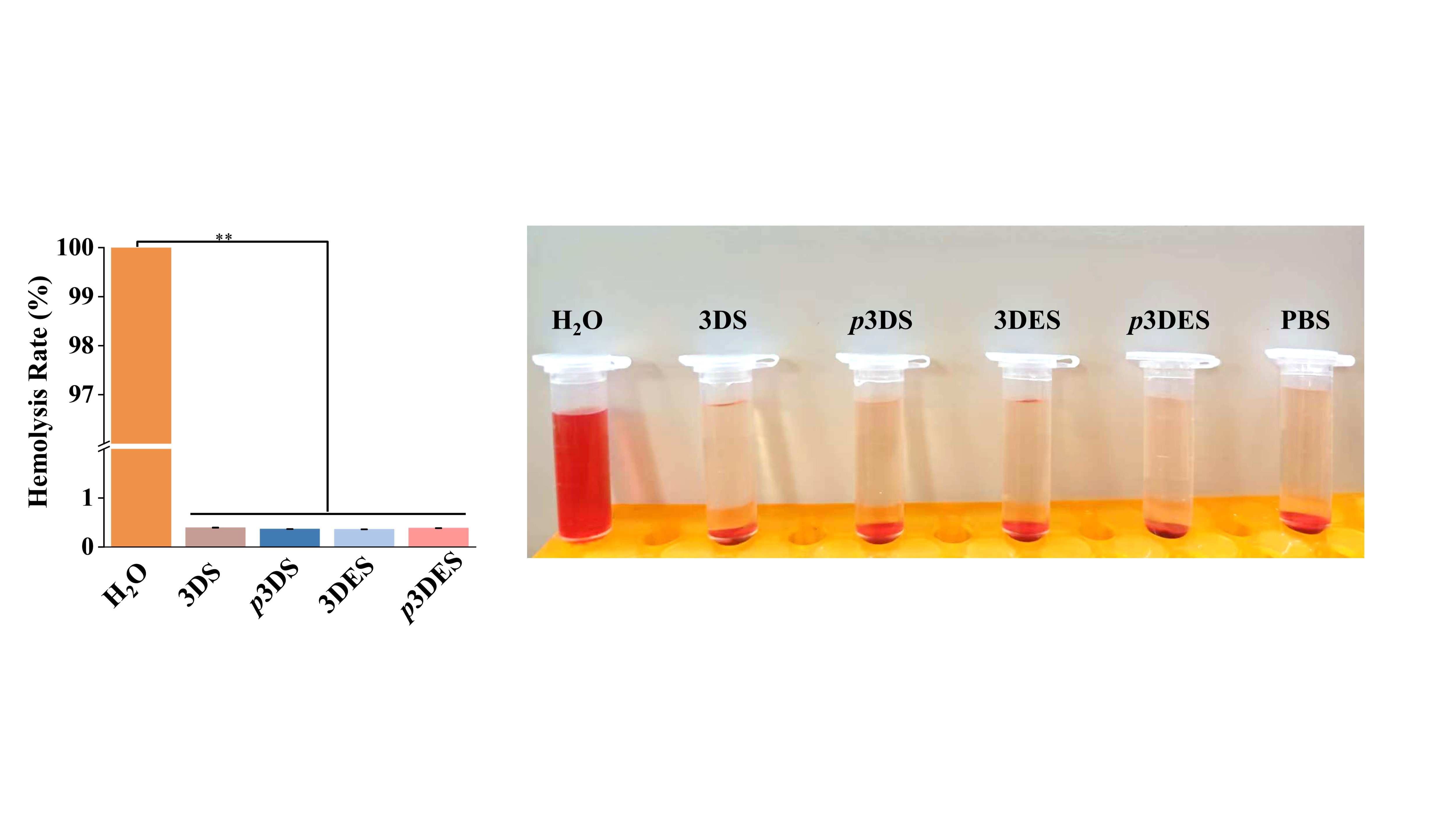


**Supplementary Figure 15**. The hemolysis test of the scaffolds. **p < 0.01, highly significant (n = 3; error bars represent standard deviation).


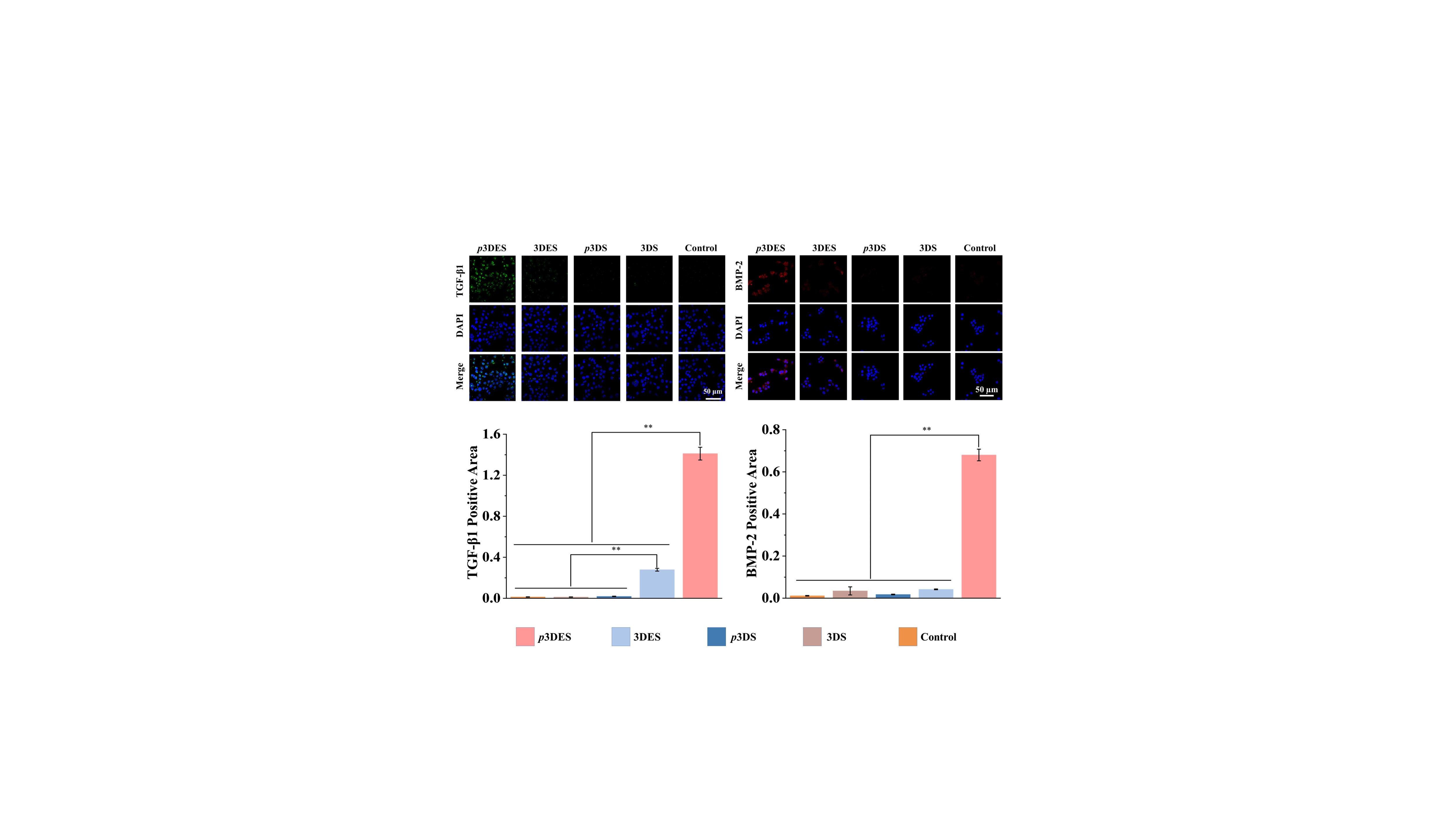


**Supplementary Figure 16**. Immunofluorescence staining results of bone-related genes (BMP-2 and TGF-β1) expression in RAW264.7 cells treated with different scaffolds for 4 days. The control group was operated without scaffold treatment. **p < 0.01, highly significant (n = 3; error bars represent standard deviation).


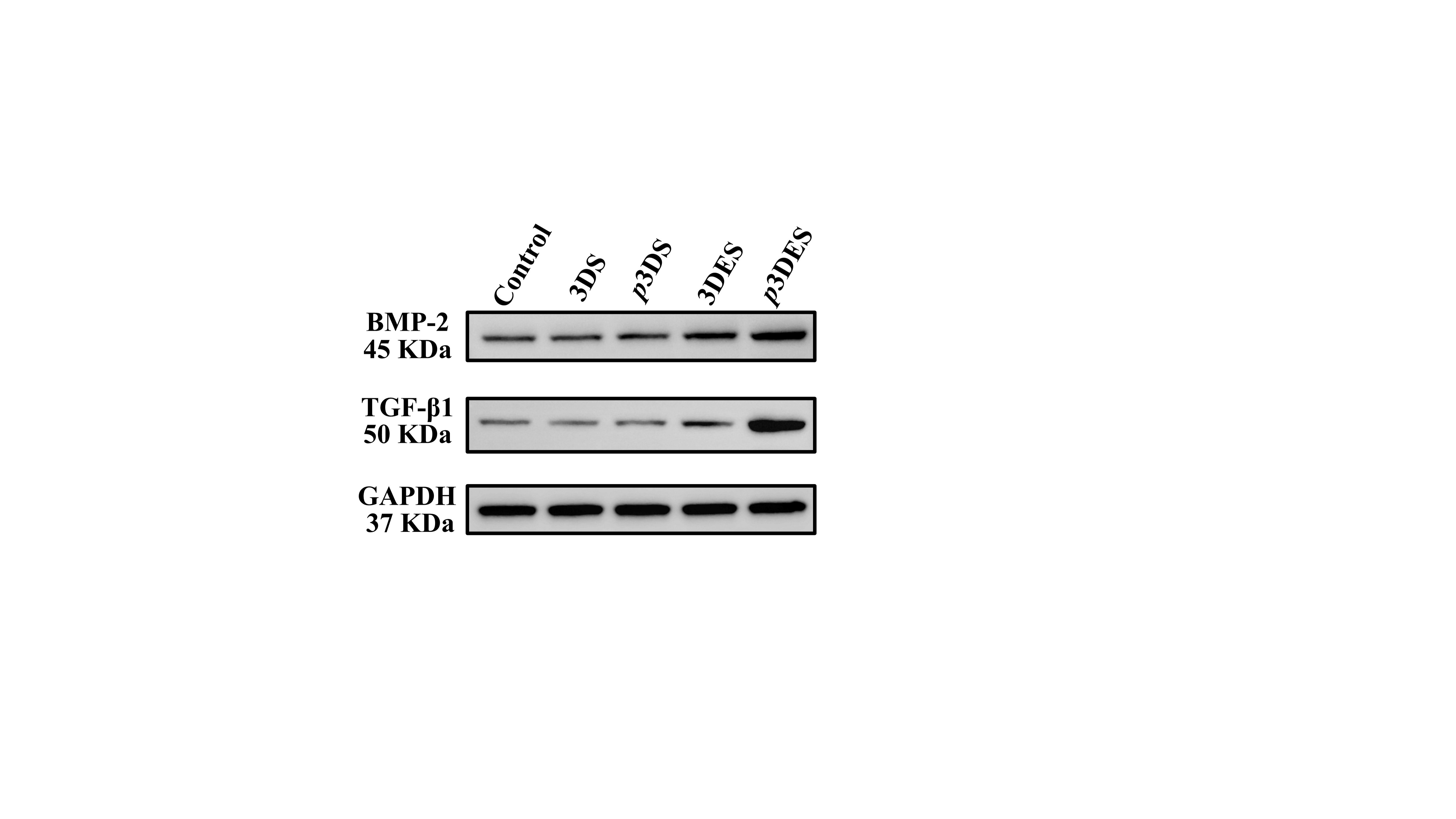


**Supplementary Figure 17**. The secretion and release of BMP-2 and TGF-β1 in macrophages RAW264.7 treated with different scaffolds. The control group was operated without scaffold treatment.


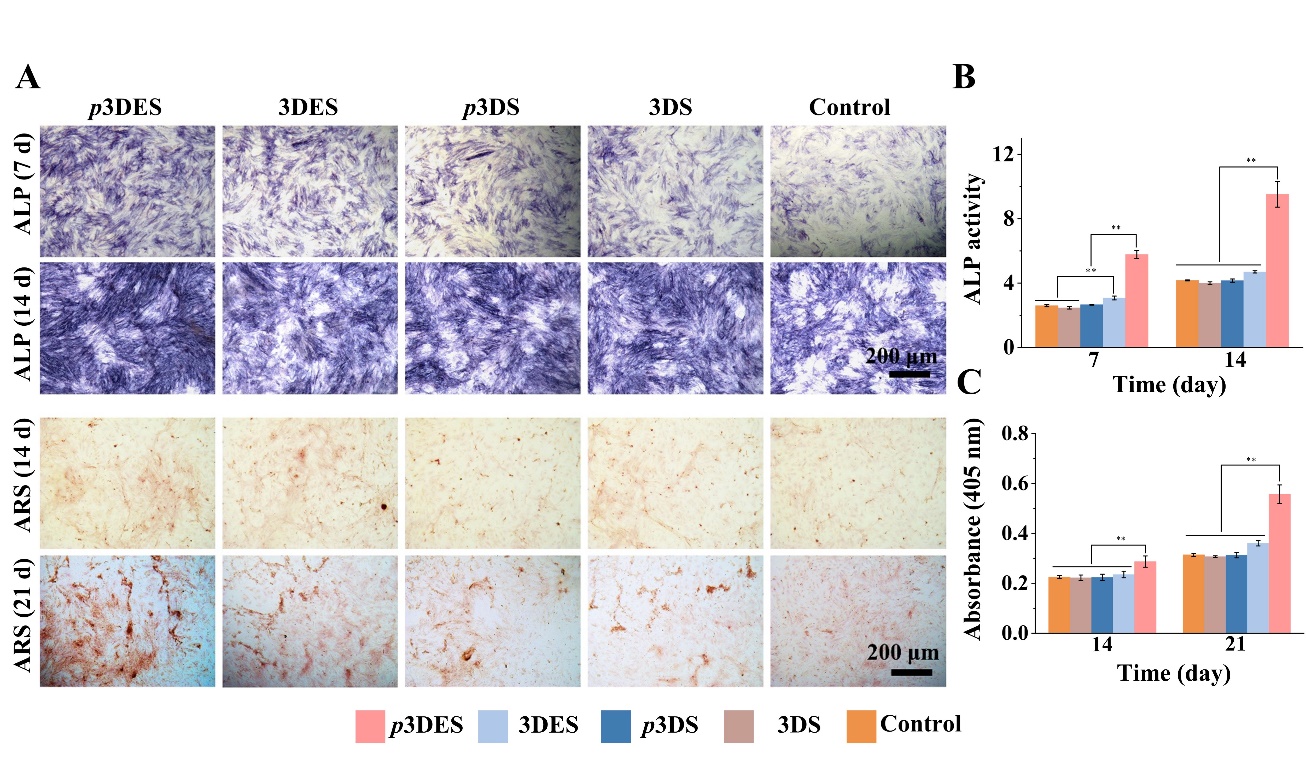


**Supplementary Figure 18**. (A) The ALP and ARS staining analysis using conditioned medium collected from RAW264.7 cells treated with different scaffolds; (B, C) The quantitative analysis of ALP and ARS staining results. The control group was operated without scaffold treatment. **p < 0.01, highly significant (n = 3; error bars represent standard deviation).


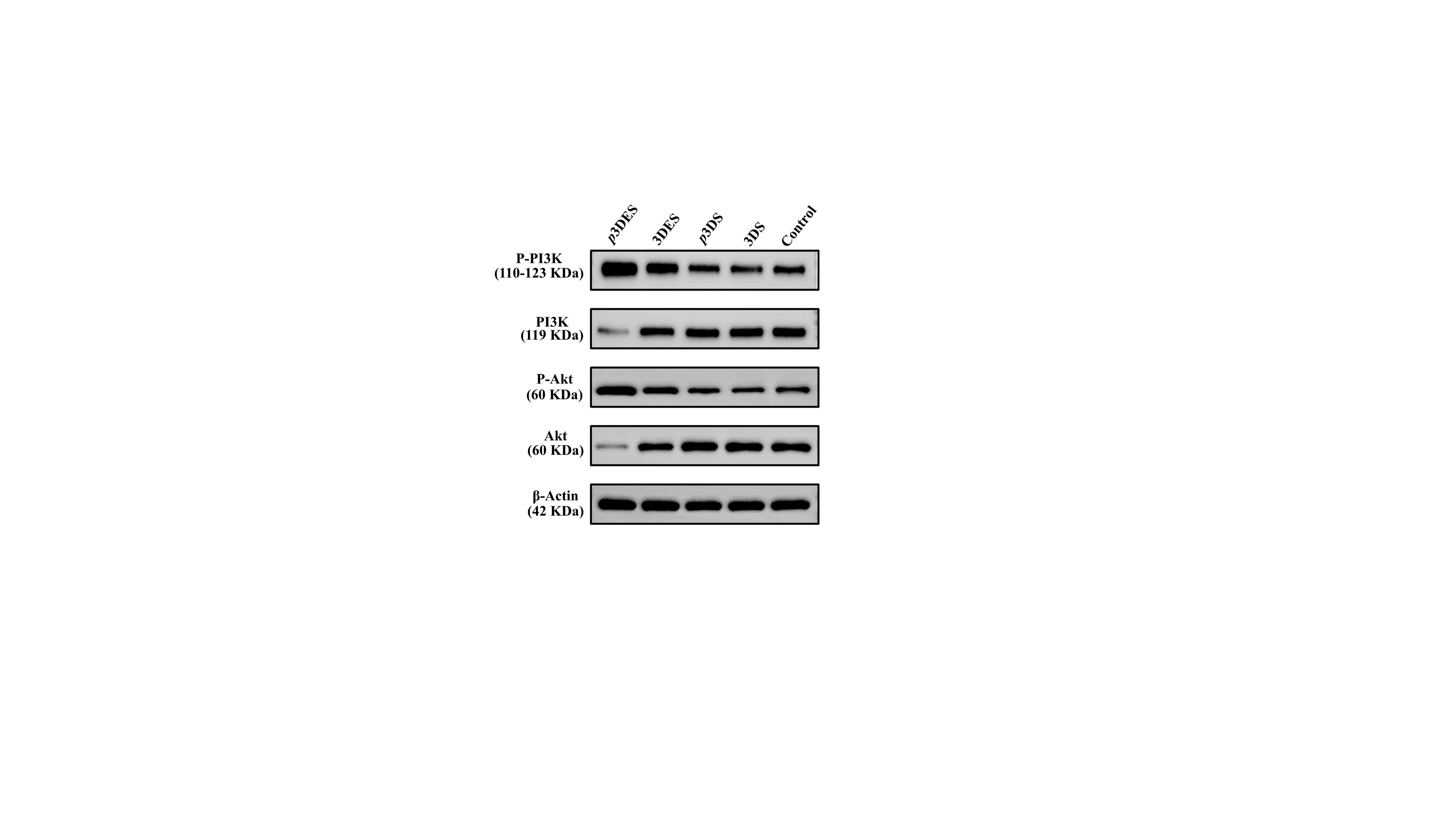


**Supplementary Figure 19**. In vitro macrophage M2 polarization mechanisms: The expression of PI3K/Akt signaling pathway-related proteins in RAW264.7 treated with different scaffolds after 24 hours. The control group was operated without scaffold treatment.


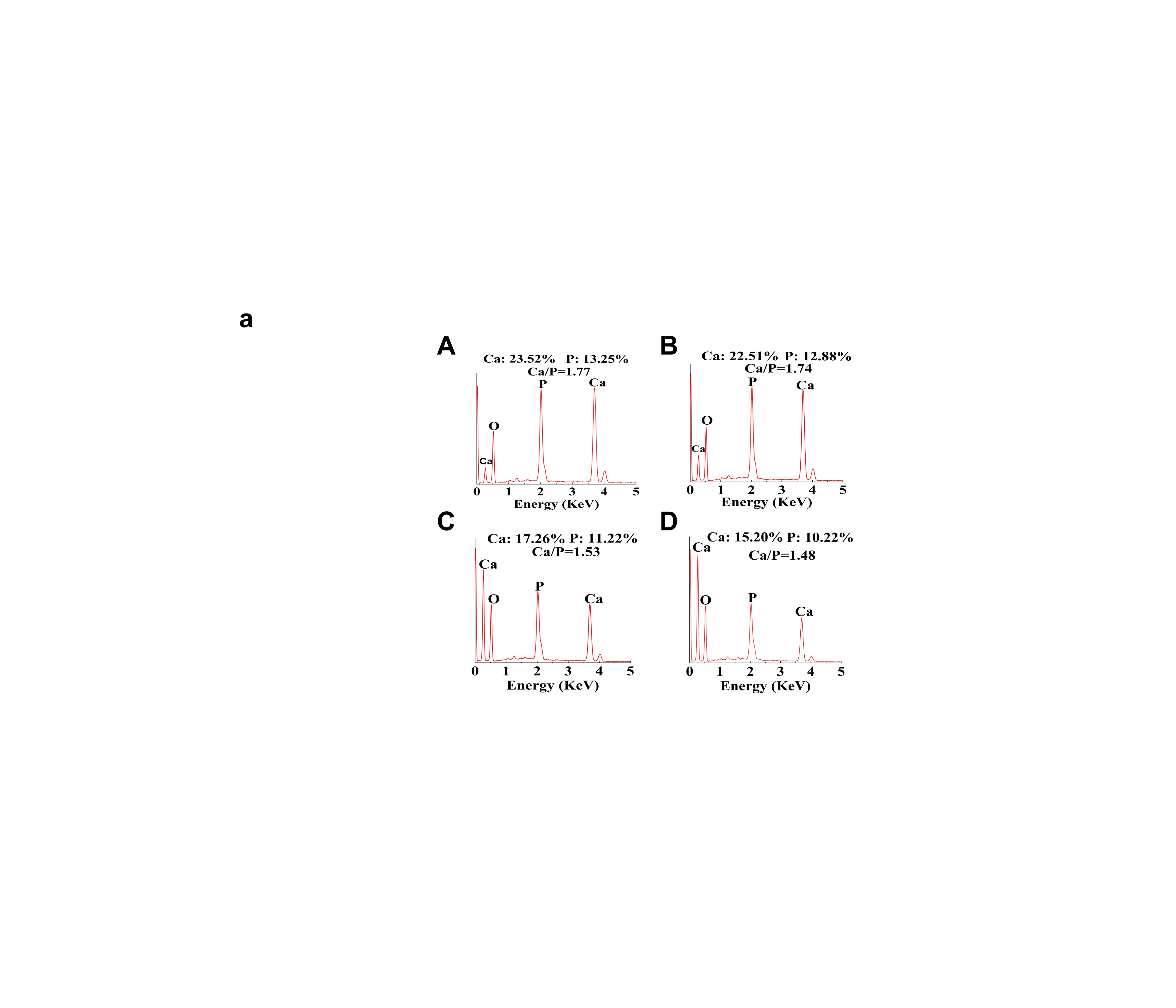


**Supplementary Figure 20**. The EDS result of surface mineralization components of polarized and non-polarized scaffolds after simulated body fluid treatment (A: *p3*DES; B: 3DES; C: *p3*DS; D: 3DS).


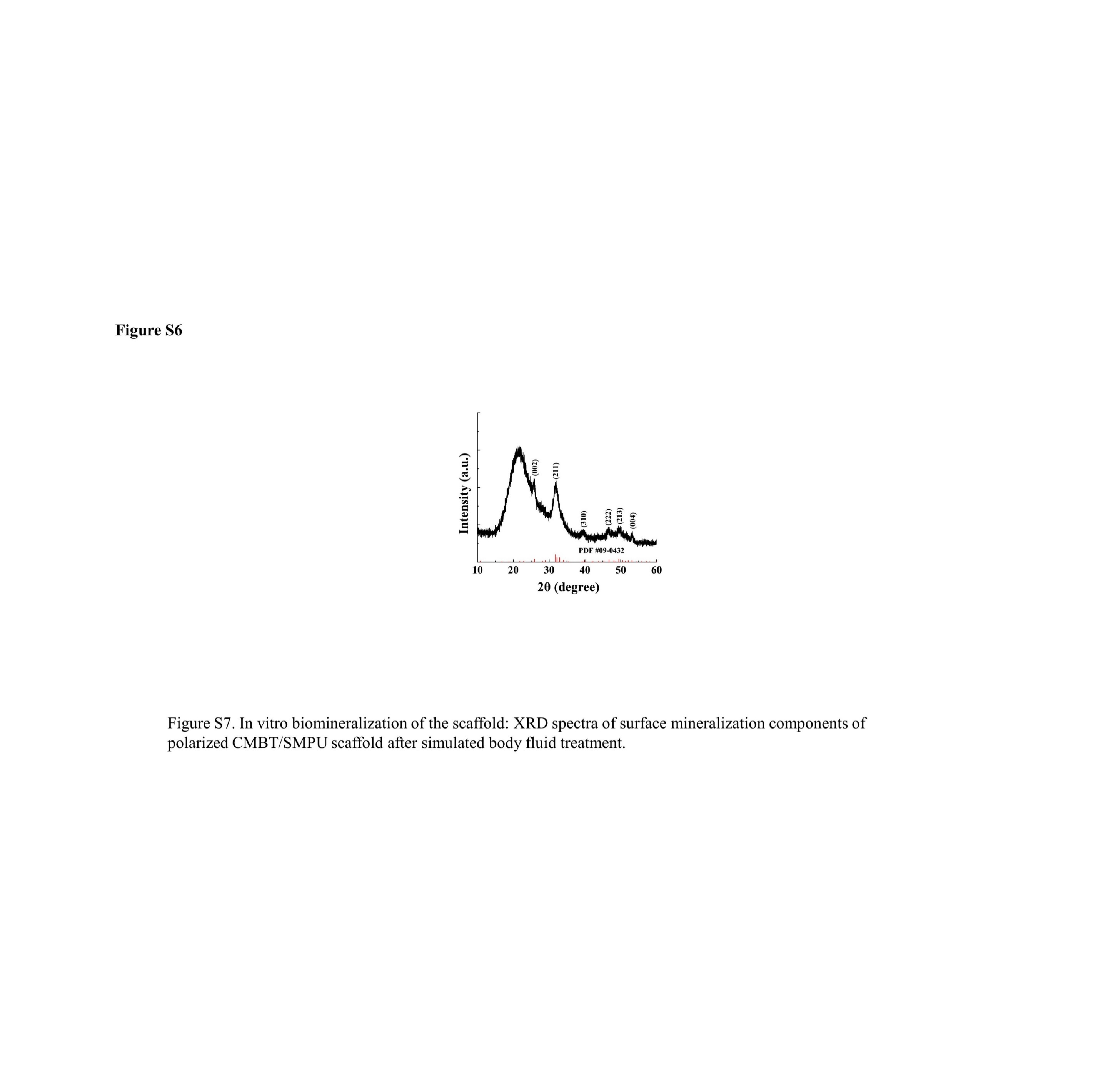


**Supplementary Figure 21**. In vitro biomineralization of the scaffold: The XRD spectra of surface mineralization components of *p*3DES after simulated body fluid treatment.


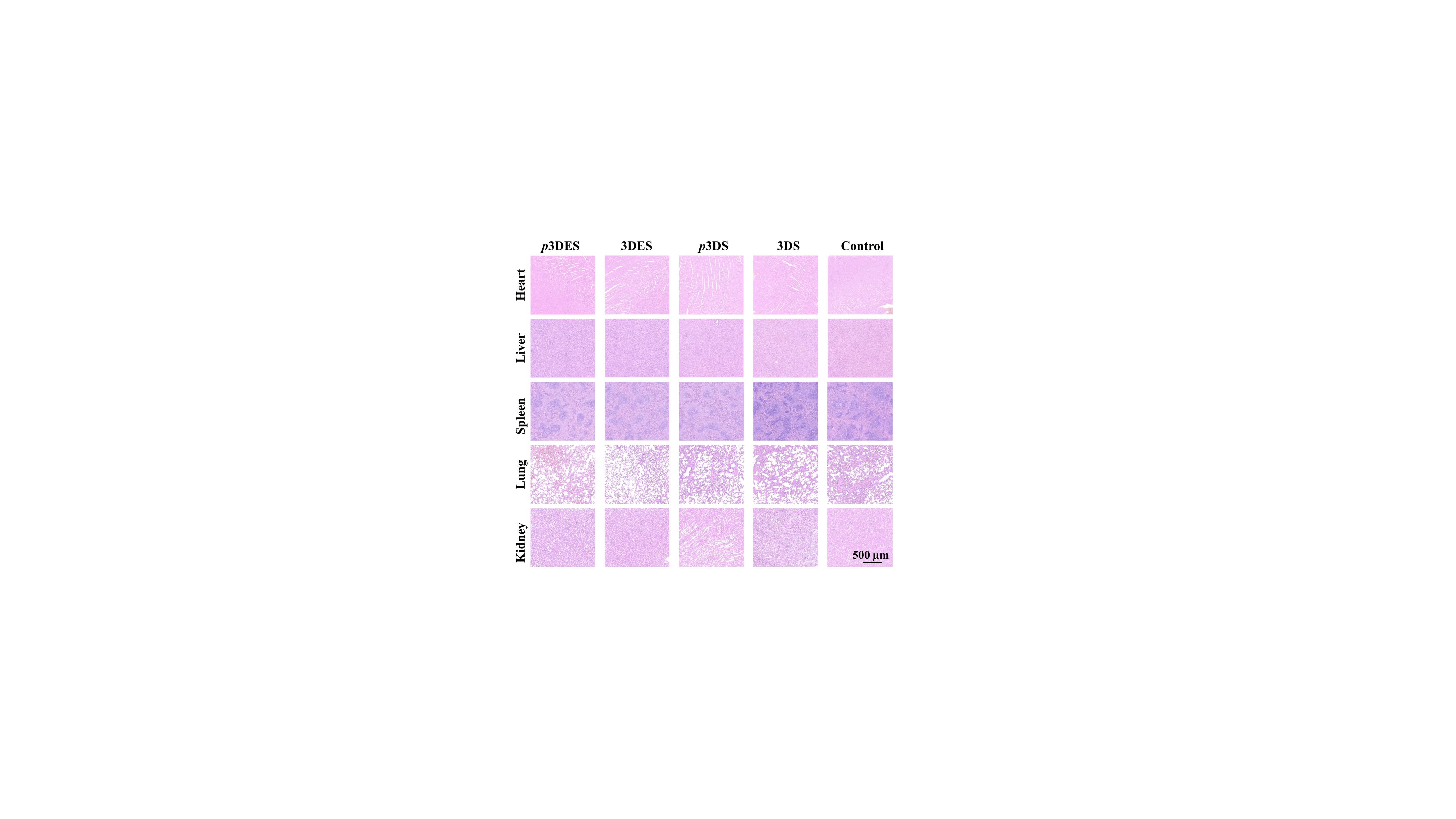


**Supplementary Figure 22**. HE staining results of heart, liver, spleen, lung, and kidney in rats after 8 weeks of scaffold implantation. The control group was operated without scaffold treatment.


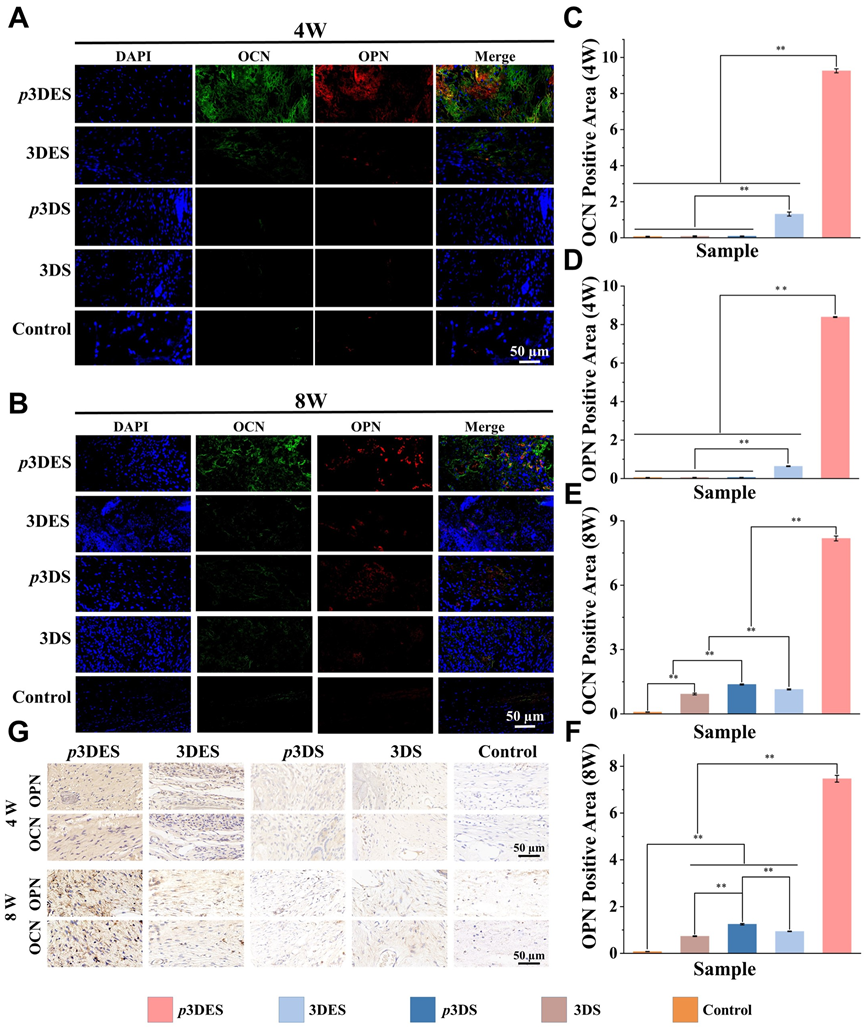


**Supplementary Figure 23**. (A-F) immunofluorescent staining of OPN and OCN in the tissues around the scaffolds at 4 and 8 weeks post-operation (blue: nucleus; green: OCN maker; red: OPN marker).; (G) immunohistochemistry staining of OPN and OCN in the tissues around the scaffolds at 4 and 8 weeks post-operation. The control group was operated without scaffold treatment. **p < 0.01, highly significant (n = 3; error bars represent standard deviation).


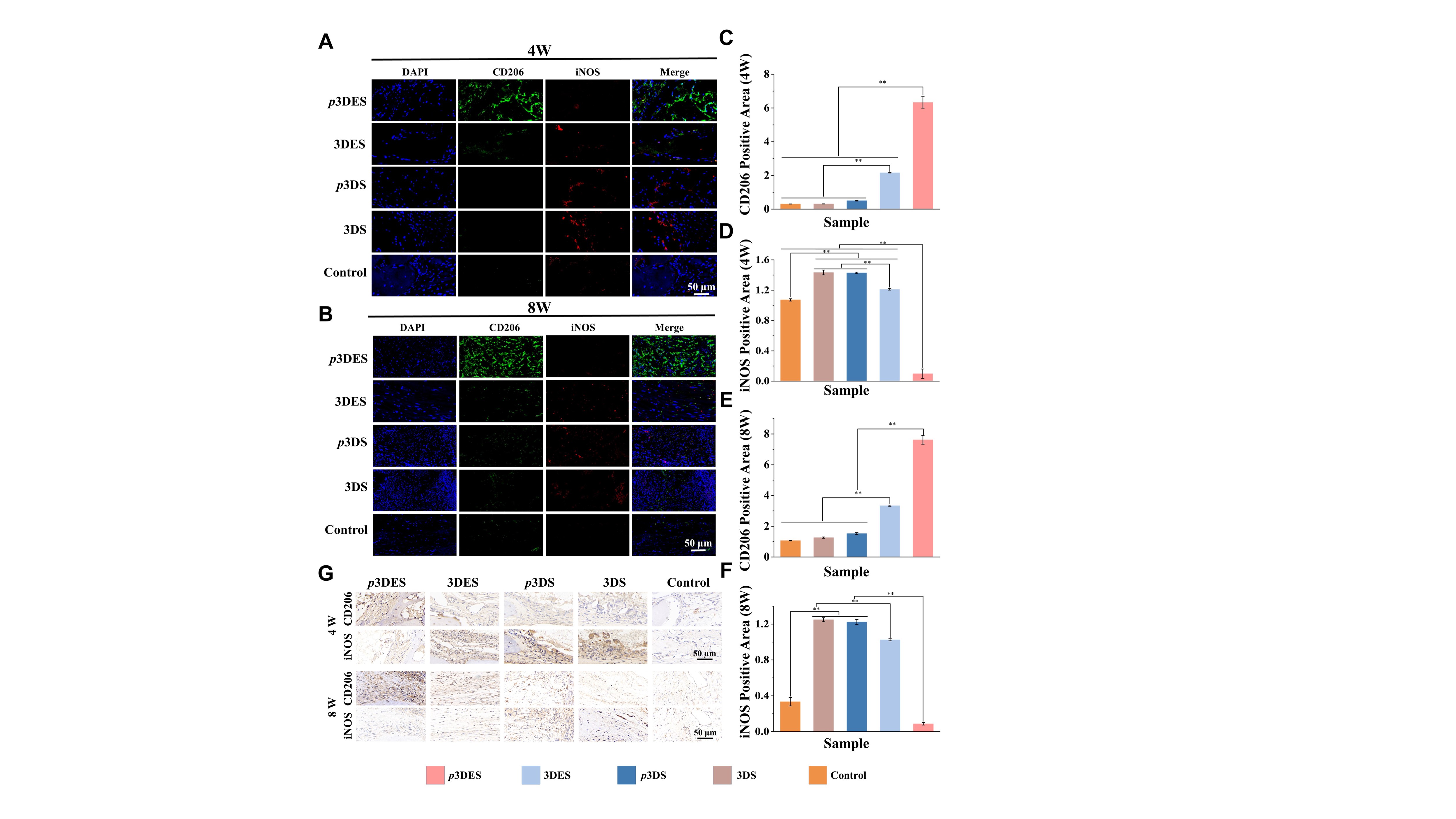


**Supplementary Figure 24**. (A-F) immunofluorescent staining of iNOS and CD206 in the tissues around the scaffolds at 4 and 8 weeks post-operation (blue: nucleus; green: CD206 maker; red: iNOS marker).; (G) immunohistochemistry staining of iNOS and CD206 in the tissues around the scaffolds at 4 and 8 weeks post-operation. The control group was operated without scaffold treatment. **p < 0.01, highly significant (n = 3; error bars represent standard deviation).

**Table 1**

Primers designed for genes related to osteogenic differentiation of BMSCs and immune response of RAW264.7 cells

| Name | Gene Forward (5′ - 3′) | Reverse (3′ - 5′) | Size |
| --- | --- | --- | --- |
| GAPDH | CTGGAGAAACCTGCCAAGTATG | GGTGGAAGAATGGGAGTTGCT | 43 |
| ALP | GACAAGAAGCCCTTCACAGC | ACTGGGCCTGGTAGTTGTTG | 40 |
| OPN | GATGAACAGTATCCCGATGCCA | GTCTTCCCGTTGCTGTCCTGA | 43 |
| OCN | CAACCCCAATTGTGACGAGC | GGCAACACATGCCCTAAACG | 40 |
| COL-1 | TCAAGATGGTGGCCGTTACT | TCTTTGCATAGCACGCCATCG | 41 |
| CD206 | TCAATGCCACTGCCATGCCTAC | AGCTTGCCGTGCGTCTTGC | 43 |
| ARG-1 | CAGCAAAGCAGACAGAACTAAG | AGAAAGGAACTGCTGGGATAC | 41 |
| iNOS | GAGACAGGGAAGTCTGAAGCAC | CCAGCAGTAGTTGCTCCTCTTC | 44 |
| TLR-2 | ACAGCAAGGTCTTCCTGGTTCC | GCTCCCTTACAGGCTGAGTTCT | 44 |
| TLR-4 | AGCTTCTCCAATTTTTCAGAACTTC | TGAGAGGTGGTGTAAGCCATGC | 47 |
| Integrin α1 | CCTGTACTGTACCCAATTGGATGG | GTGCTCTTATGAAAGTCGGTTTCC | 48 |
| Integrin α2 | CACAGTTCATTTTTAGGTTACT | CACATTGCCATGCTTGTTAACA | 44 |
| Integrin α5 | ACAGTTCGAGCCCATGGCT | CTGAACACATTCTTTATGCTC | 40 |
| Integrin β1 | CTACTGGTCCCGACATCATCC | TGACCACAGTTGTCACGGCAC | 42 |
